# A future for digital public goods for monitoring SDG indicators

**DOI:** 10.1038/s41597-023-02803-x

**Published:** 2023-12-07

**Authors:** Dong Liang, Huadong Guo, Stefano Nativi, Markku Kulmala, Zeeshan Shirazi, Fang Chen, Gretchen Kalonji, Dongmei Yan, Jianhui Li, Robert Duerler, Lei Luo, Qunli Han, Siming Deng, Yuanyuan Wang, Lingyi Kong, Thorsten Jelinek

**Affiliations:** 1 International Research Center of Big Data for Sustainable Development Goals, Beijing, 100094 China; 2 Key Laboratory of Digital Earth Science, Aerospace Information Research Institute, Chinese Academy of Sciences, Beijing, 100094 China; 3 University of Chinese Academy of Sciences, Beijing, 100049 China; 4 National Research Council of Italy, Institute of Atmospheric Pollution Research – Unit of Florence, Roma, Italy; 5 Institute for Atmospheric and Earth System Research, University of Helsinki, P.O. Box 64, 00014 Helsinki, Finland; 6 Sichuan University-The Hong Kong Polytechnic University Institute for Disaster Management and Reconstruction, Chengdu, 610207 China; 7 Computer Network Information Center, Chinese Academy of Sciences, Beijing, 100083 China; 8 State Key Laboratory of Remote Sensing Science, Aerospace Information Research Institute, Chinese Academy of Sciences, Beijing, 100101 China; 9 Integrated Research on Disaster Risk, International Science Council, Beijing, 100094 China; 10 Taihe Institute, Beijing, 100022 China

**Keywords:** Policy, Environmental impact

## Abstract

Digital public goods (DPGs), if implemented with effective policies, can facilitate the realization of the United Nations Sustainable Development Goals (SDGs). However, there are ongoing deliberations on how to define DPGs and assure that society can extract the maximum benefit from the growing number of digital resources. The International Research Center of Big Data for Sustainable Development Goals (CBAS) sees DPGs as an important mechanism to facilitate information-driven policy and decision-making processes for the SDGs. This article presents the results of a CBAS survey of 51 respondents from around the world spanning multiple scientific fields, who shared their expert opinions on DPGs and their thoughts about challenges related to their practical implementation in supporting the SDGs. Based on the survey results, the paper presents core principles in a proposed strategy, including establishment of international standards, adherence to open science and open data principles, and scalability in monitoring SDG indicators. A community-driven strategy to develop DPGs is proposed to accelerate DPG production in service of the SDGs while adhering to the core principles identified in the survey.

## Introduction

Growing accessibility to digital technologies and their integration into the daily lives of billions of people has led to rapid transformations in modern societies, including: improving communication, introducing new business opportunities while better facilitating existing enterprises, offering greater access to educational opportunities, and improving access to information and services related to finance, investment, health care, and other essential and recreational amenities^1^. This digital transformation has also opened up new opportunities for progress on multinational collaboration in addressing the risks and challenges that we face collectively. With an emphasis on science, technology, and innovation, the United Nations (UN) is working to improve multi-stakeholder cooperation for technological innovation to achieve the Sustainable Development Goals (SDGs) and to encourage multilateral coordination between stakeholders through its Technology Facilitation Mechanism (TFM). Realizing the importance of digital technologies within this context, the UN Secretary-General convened a High-level Panel on Digital Cooperation in 2018 to advance proposals to strengthen cooperation in the digital space among governments, the private sector, civil society, international organizations, academic institutions, the technical community, and other relevant stakeholders. The report by this High-level Panel not only influenced the content later released in the Report of the Secretary-General’s Roadmap for Digital Cooperation, but also elaborated upon the issues surrounding the digital sphere. The report recommended the promotion of an inclusive digital economy and society, digital public goods (DPGs), digital inclusion, improving digital capacity, establishing digital human rights, responsible applications of AI, digital trust and security, and global digital cooperation^2–4^.

The increasing emphasis on digital technology is due to its growing influence and rapid adoption, driven by its potential to enhance economic growth and by the value it can provide for companies and communities pushing rapid development of necessary infrastructure and services^5^. This value is partially due to the growing volume of valuable data produced daily as a result of human interactions with digital technology, and partially due to its use across a wide range of digitized services that provide enormous potential for both commercial and research purposes^6^. Understandably, the growing volume of digital data is also highly desirable and useful for developing a consensus on causes, drivers, impacts and trends of sustainability challenges in various communities worldwide. Digital knowledge systems, if open and freely accessible, can also support the implementation of solutions that are traceable, verifiable, and quantifiable by the scientific community across the world. However, despite all advancements in digital technologies, the understanding of how these digital resources can serve the 2030 Agenda for Sustainable Development and the SDGs remains abstract. To move this discussion forward, this paper explores the question *how can the global community promote consensus and develop new mechanisms to best utilize digital resources to achieve the SDGs?*

In the UN Secretary-General’s Roadmap for Digital Cooperation, the concept of DPGs provides a useful mechanism to address the translation of raw, multi-source data into actionable information for policy and decision making. DPGs generally include datasets, software, and other digital content made openly accessible to the public. However, as there does not yet exist a universally accepted definition for DPG, there are attempts to create a clearly defined standard for what constitutes a DPG^7^. The term DPG stems from the existing economic concept of public goods and its derivation, global public goods (GPG). In a broader sense, public goods are defined as goods that are accessible to all without exclusion or inhibition^8^. From the more traditional public goods of massive infrastructure, such as national highway systems and national parks, to more recent ones like television networks and internet infrastructure, the gradual trend of digitalization and globalization represents society’s adaptation to new technologies^9,10^.

GPGs have been an influential apparatus for the international community, especially for the UN, to push for universally beneficial initiatives such as the Millennium Development Goals (MDGs). Similarly, there is tremendous potential for DPGs to facilitate the implementation of the 2030 Agenda for Sustainable Development. However, unlike the extensive body of work available on GPGs, concepts regarding DPGs are still in the process of development^11,12^. The term “digital” refers to resources that can be created, modified, shared and made accessible by means of digital or information technologies, which can also be communicated through the IoT (Internet of Things) infrastructure^13^. What makes a digital service or resource a “public good”, however, is still under debate.

The Digital Public Goods Alliance (DPGA) has made significant contributions in promoting the concept of DPGs and their accessibility, particularly in low- and middle-income countries^7^. As a broad multi-stakeholder alliance, DPGA has established a platform for sharing DPGs and defined a set of nine indicators as a benchmark for defining five categories of DPGs: 1) open AI models, 2) open content, 3) open data, 4) open software, and 5) open standards. The nine defined indicators drafted by the DPGA are: a) relevance to SDGs, b) use of approved open licenses, c) clear ownership, d) platform independence, e) documentation, f) mechanisms for extracting data, g) adherence to privacy and applicable laws, h) adherence to standards and best practices, and i) safe, private, secure, and legal content. These indicators offer a sound intellectual and functional foundation to promote open science and improve the quality and accessibility of DPGs^14^. They are also in alignment with the principles of open science recently agreed upon by the member states of the United Nations Educational, Scientific and Cultural Organization (UNESCO)^15^. However, it has been reasoned that these definitions equate free and open-source software and services with DPGs and promote production aspects (design, development, and distribution) rather than adoption and appropriation to match local demands^14^. The indicators for the SDGs, internationally accepted within the UN, describe benchmarks to ensure quality, accessibility, privacy, and security. These are quite useful at an organizational and institutional level for implementing and facilitating sustainability projects. However, they have not always proved to be functional at different levels of governance for policy development processes or administrative oversight. The same can be said when they have been used internationally to measure the progress made and the levels of sustainability achieved, due to the diversity of choices among DPGs and the operational and institutional standards adopted by the various subjects involved. Therefore, in the context of the SDGs, in addition to facilitating the development, accessibility, and discoverability of DPGs, there is also a pressing need to identify and build consensus concerning the most relevant and effective options that are currently available^16^. Further exploring the relationship between DPGs and SDGs will create opportunities to realize the potential of DPGs.

The current progress in innovative technologies, the growing role of big data, and the continuous improvement of Earth observation platforms and technologies all promise to support the critical role of DPGs for SDGs by providing instruments to monitor the SDG indicator framework and to produce data for policy and decision support systems^17–19^. The global SDG indicator framework provides a foundation for quantifying SDG progress through monitoring social, economic, and environmental dimensions, as well as a means to demonstrate interdependency and synergy among these various dimensions of sustainability^20^. SDG indicators provide an important means to inform national-scale policies and support development of implementation strategies and allocation of resources. In recent years, the use of big data based on comprehensive observations to pursue the SDGs has begun to be considered a valuable option^21^. The information generated on the five Earth systems (geosphere, biosphere, cryosphere, hydrosphere, and atmosphere) has significant value and, once data concerning these multiple interacting systems is made increasingly accessible to the public, the information will stimulate public awareness, knowledge, and research in order to encourage innovative solutions for globalization and regional challenges on a local level^22^. This concept is also fully in line with the efforts of the United Nations Environment Programme (UNEP) to promote environmental data as a DPG. Unfortunately, the lack of access to qualified and timely datasets and methodologies hinders progress, particularly due to the added complexities of uneven development and capacity challenges between different regions and countries^23^. Therefore, there is a need for mechanisms (e.g., software models and tools) to develop simplified information products delivering standardized information for uniform adoption and wider acceptability for each spatial scale. This will strengthen the ability of public systems to adopt and utilize this information in their policy and decision development processes.

While DPGs are required to be published as open data, open software, and open computational infrastructure and services, DPGs in the context of SDGs also include standardized, scientific, and credible mechanisms. These mechanisms give the necessary context and understanding when framing justifiable actions that ensure sustainability with the best possible synergy between the three dimensions of the SDGs (economic, social, and environmental)^15^. Like existing public goods (roads, communication networks, etc.) that provide access and services to all, DPGs for SDGs should ensure accessible mechanisms and resources to convert multi-source data to information. Moving forward, it is therefore necessary that DPGs for SDGs are developed using innovative data sources and methods based on the principles and standards established by the DPGA, while at the same time focusing on mechanisms for wider acceptability, compatibility, and adoption by communities allowing for comparable and multi-scale monitoring of SDGs around the world^24–28^.

In recognition of the particular needs of DPGs related to sustainability research, the International Research Center of Big Data for Sustainable Development Goals (CBAS) develops SDG data infrastructure that heavily relies on and promotes the concepts of open science and DPGs, such as the SDGSAT-1 satellite and the SDG Big Data Platform. Considering the inherent complexity of multisource data integration and analysis, as well as the challenges in communicating actionable information to policy and decision-making systems, CBAS identified DPGs as an important means to facilitate information-driven policy and decision-making processes for SDGs^29^. CBAS organized an expert survey to consult with researchers and professionals within its network of collaborators specializing in the fields of Earth observation, Digital Earth, and big data as a consultative exercise to identify core principles and possible key actions to guide and inspire new ideas and concepts in future research and capacity development efforts toward evaluating and assessing SDG indicators^29–31^.

A detailed methodology of the survey and analysis of the results is provided in Section 5. In summary, the authors reached out to 300 experts from around the world based on their participation in relevant conferences and analyzed the 51 survey responses received to form the recommendations and conclusions presented in this article. In addition to the stated objectives of formalizing core principles and key actions, the authors also discuss and propose a cooperative framework with a participatory approach to developing DPGs based on the core principles identified within the survey.

This survey provides an initial effort to develop a broad framework for engagement with other relevant organizations and stakeholders to deliberate on practical opportunities for the development of DPGs for SDGs to support policy and decision-making processes.

## Results

Most of the international experts who were consulted agreed in principle with the premise that DPGs can and will help the achievement of SDGs. However, they also pointed out that this achievement is not unconditional and guaranteed, with about 13% of respondents believing that DPGs might only conditionally advance the SDGs. The answers highlighted the factors necessary to ensure a correct identification and evaluation of DPGs: the definition of concepts, the recognition of initiatives and experiences, and the role of organizations, cooperation, and political support. Only after consideration of these factors can the DPGs be reliably transformed into a common tool and be made accessible for wider dissemination for the SDGs. Otherwise, SDG research may not make progress at the pace which the funding allows. This was particularly cited as a necessary condition for the successful deployment of DPGs for SDGs. Implementing this concept will represent a massive global big data enterprise. Ensuring multi-stakeholder commitment and promoting solidarity through a well-managed cooperation mechanism were also considered important to guarantee continued support for a global program on DPGs for SDGs.

Several important resources were recognized to be relevant to DPGs for SDGs. Due to the location-based attributes of SDG targets and their indicators, Earth observation data and geographic information (along with their respective software, models, standards, and technologies) were indicated to be important digital resources for a rapid implementation of SDGs at global, regional, and local levels.

A good example of such resources is represented by Big Earth Data, which provides essential context to enable professional and political decision-making toward implementing the SDGs^32,33^. As such, about 35% of experts specifically cited Earth observation, remote sensing, or Big Earth Data as key assets. The relevance of mobile data for mapping human activity was also stressed as a key data resource due to the high volume of mobile devices in use throughout the world. As suggested by one of the respondents, “Science is now big data centric.” Therefore, big data, AI models, and several variants of these digital resources and methods were widely considered to be important instruments that can fuel the development of DPGs^34^. Finally, a minority of experts have proposed, as essential sources of information, *in-situ* observational data and (experimental) model-generated data to simulate soil, atmosphere, and ocean processes utilizing deep learning techniques.

Overall, the experts expressed strong support for open data, open databases, open data models, and open-source software; these keywords are present in about 63% of the answers relating to core digital principles. FAIR (Findable, Accessible, Interoperable, and Reusable) data principles also had strong support among respondents, and FAIR was specifically referenced several times^35^. Experts considered scientific and data infrastructures (such as data cubes and high-performance computing resources) as important assets and digital technologies that are necessary for the development of high-quality DPGs and the facilitation of the rapid implementation of the SDGs globally. Several respondents also highlighted the importance of multi-source data and data interoperability approaches to generate and advance digital resources that are useful for achieving the SDGs globally. These digital resources can then be complemented with open software to promote innovation.

However, experts also stressed that the most important aspects of ensuring the success of any DPG initiative are the will and ability to act. As an example, to determine the effectiveness of a DPG initiative, it was suggested to consider the following factors: the level of access, the tools available, and the users’ skills. Around 23% of the experts cited education and training as a key action or challenge to move forward with DPG implementation and overcome existing barriers. Similarly, the allocation of financial resources was cited as another critical limitation. While the digital technologies and resources required to facilitate DPG development are increasingly available, current limiting factors include the shortage of funding, data sharing, and access to scientific modeling infrastructures. These issues have limited the pace of progress, hurting developing countries in particular. The respondents also recognized that legislative, geo-political, socio-economic, and cultural challenges have hindered progress. Along the same lines, some respondents argued that isolated activities have produced duplicated actions at the cost of time and resources. Therefore, coordinated efforts across administrative boundaries were considered highly desirable. With this in mind, the need to improve the capacity of developing countries to address multinational and coordination challenges was stressed. In particular, investments are needed to improve the knowledge and ability to acquire data from multiple sources, to use analytical tools, and to access computational models to facilitate this process.

### Defined core principles and key actions

With their responses, the experts identified several core principles and a broad range of key actions. Among the proposed principles, the most commonly stressed point was the need to ensure that information and data are accessible to the public to promote awareness, knowledge, research, and innovation and to encourage innovative solutions to global and regional challenges. This principle was indicated in about 70% of the answers. Table 1 lists the core principles along with a set of associated key actions, which were defined by the experts to facilitate and guide the development process of DPGs for implementing SDGs. The principles and actions shown in the table are the result of synthesis and grouping operations carried out by the authors of this article.

**Table 1:** Core Principles and Possible Key Actions as suggested by the experts for developing DPGs for SDG indicators.

Core Principles	Possible Key Actions
1 Universality of science	1.1 Promote open science, open data, and open knowledge.
	1.2 Encourage scientific partnerships, collaborations, cooperation, and multi-disciplinary stakeholder engagements.
2 Scalability	2.1 The development process of DPGs for SDG indicators should incorporate multi-stakeholder engagement and a mechanism for a bottom-to-top approach.
3 Inclusive and innovation-driven development process	3.1 DPGs for SDG indicators should be initiated as community-supported open-source projects to drive innovation and creativity.
	3.2 Create global SMART (Specific, Measurable, Achievable, Realistic, and Timely) objectives, aligning with national goals.
	3.3 Ensure participation and engagement of relevant stakeholders from developing countries and local communities.
	3.4 Identify and engage projects and organizations already developing similar or related digital products to avoid duplication.
	3.5 Promote interdisciplinary collaborations and pooling of digital and human resources between participants, leading organizations, and public sector institutions.
4 Availability and accessibility to all without restrictions	4.1 An open online distribution platform for DPGs for SDG indicators supporting information and application infrastructure should provide access to the necessary tools, resources, and data used in the development of DPGs.
	4.2 Intuitive and friendly user-interface for wider adoption and public awareness.
	4.3 Compliance with FAIR principles.
5 Quality, acceptability, impartiality, and reproducibility	5.1 A system of quality control, standardization, and accreditation through an expert oversight committee with the support of a UN custodian agency should be set up.
	5.2 Ensure and maintain effective data standards and regulations.
	5.3 Highlight usability through case studies, pilot projects, and demonstration studies.
	5.4 Develop a mechanism for user support and documentation to attract adoption and utility.
6 Enhanced training and upskilling across sectors/nations	6.1 Educational support to promote development of skills at all levels.
	6.2 Promote inspirational content to motivate younger generations to contribute their expertise to this public process in the future.

## Discussion

In the past decade, the availability and knowledge of digital tools and resources have rapidly improved worldwide. Nevertheless, the differences in capacity and priorities will likely be a problem for any development process working to produce DPGs for SDG indicators, which are intended for global use. The survey results highlighted that more than 30% of the experts believe a single program or a single agency is not in a position to facilitate the development of the diverse range of DPGs necessary for generating SDG indicators. Rather, this endeavor requires international and multi-stakeholder cooperation. Based on these opinions, the authors developed a consensus on the need for a cooperative framework applying a participatory approach that should observe the principles of the universality of science in accordance with the first Core Principle identified in Table 1.

Considering Key Actions 1.1 and 1.2, which promote open science, inclusivity, consultations, joint contributions, and shared benefits, this paper suggests a community-supported open-source development approach that will improve innovation and novelty in methods and processes. Supported by a UN-driven recognition program, talent at all skill levels could be encouraged to participate in this community-led development process.

The community approach provides a multi-stakeholder development process that not only provides a strong process to identify, sort, and develop mainstream DPGs for SDGs, but also creates an opportunity to hear from stakeholders from different scales and perspectives. If properly managed, it has the potential to also address the paradoxes identified regarding scaling challenges of DPGs^36^. The community-based approach also supports the idea of a continual development process that enables new ideas to flourish and to be recognized.

**Fig. 1:**
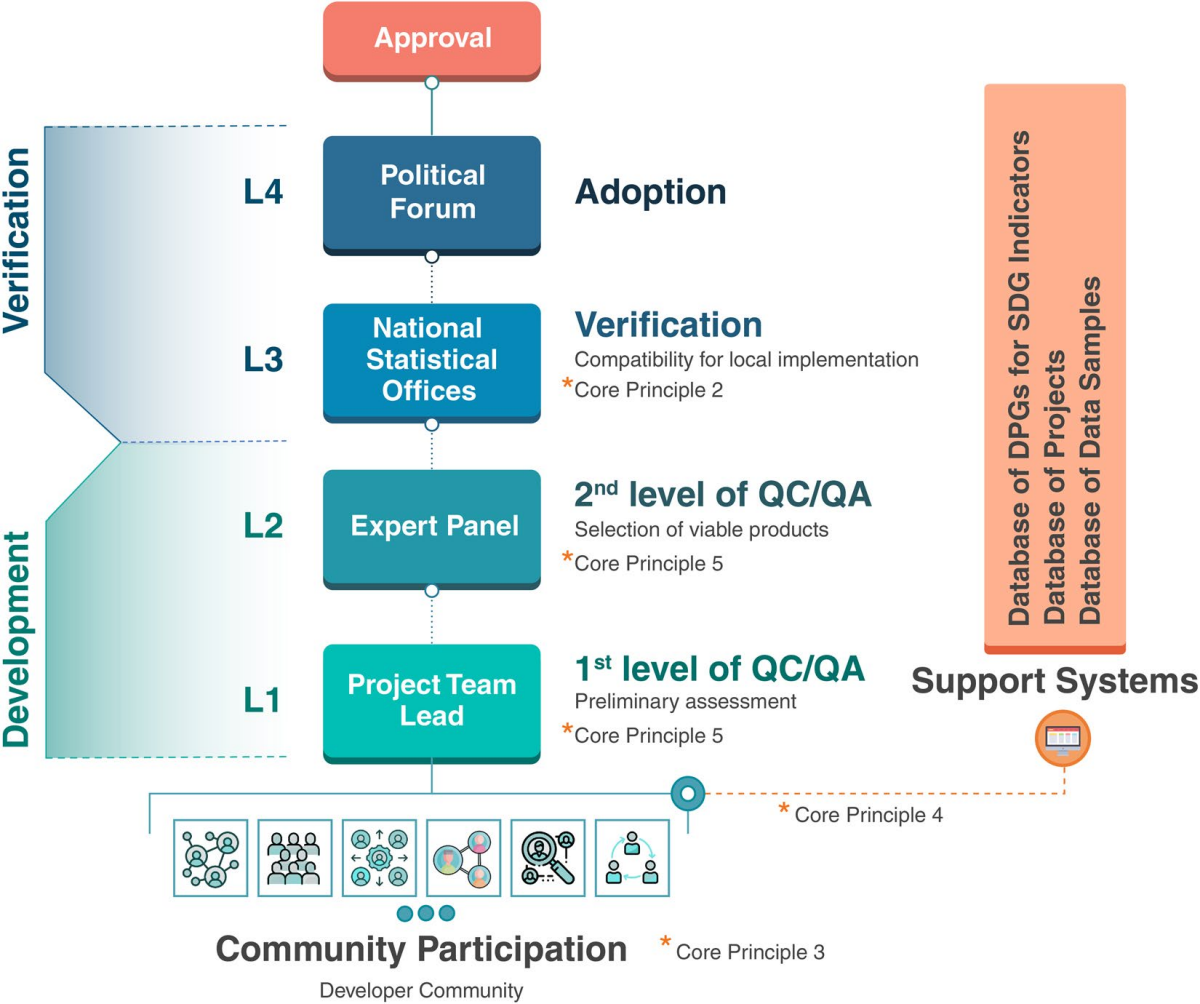
Proposed community-driven open-science process to develop DPGs for SDG indicators.

A more detailed, tentative flowchart of the proposed process is provided in Fig. 1. The current framework is by no means complete and devoid of any flaws, but nevertheless there is strong potential for using this process to create mechanisms for supplying and scaling DPGs to different communities globally. The following section highlights important challenges that were identified by the authors during discussions and provides suggestions on the key actions to address these challenges.

### Organizational challenges

DPG development that applies the open-science paradigm and is supported by the DPG community at large would be an attractive solution for addressing many implementation issues. This kind of governance scheme would provide the necessary bottom-up approach that is suggested by Key Action 2.1 of Table 1 (Core Principle 2). In addition, it would help encourage highly talented data scientists, programmers, and digital technology experts to voluntarily participate in devising novel solutions, as indicated by Core Principle 3. However, organizing and streamlining these efforts could prove very challenging. In the absence of a designated team, an open-source development environment could lead to competing methods and products. Addressing this challenge will require efficient Quality Assurance and Control (QA/QC) mechanisms (Core Principle 5). Similarly, a community-based development approach could improve the efficiency of the development process, particularly one in which expert volunteers collaborate with research and academic institutions to broaden the scope of development against defined goals and deadlines.

Some of these governance challenges could be solved by appointing product leaders for the separate classes of DPGs as defined by DPGA (i.e., models, content, data, software, and standards). These managers could be designated by the various agencies that manage and maintain the different SDG indicators. The designated product leaders would work as focal persons for collecting community input, while having a clear understanding of the SDG indicators at hand and the related auditing requirements. Finally, they might help organize the community development process, as per Key Action 3.2, and provide relevant guidance to community developers and serve as a preliminary QA/QC. Subsequently, in a second phase of the development process, a “Committee of Experts” could be established to evaluate the different digital products received and act as a higher level of QA/QC in keeping with Key Actions 5.1 and 5.2. The Committee of Experts could select a list of viable products to be tested and verified by National Statistical Offices (NSO) for compliance and applicability (see Fig. 1). In addition, they could help achieve Key Action 5.3. After verification from NSOs, the product could be adopted through a designated political forum for SDG evaluation reporting purposes by interested countries and organizations.

In the development process discussed, however, there are possibilities for duplications of effort. Acting on Core Principle 4 (Key Actions 4.1, 4.2, and 4.3) will provide the necessary resources and information to avoid possible duplications. A descriptive and open catalog of ongoing developing efforts should help potential developers understand which ideas are truly innovative, thus saving time and avoiding unnecessary effort.

An important organizational challenge will be to fund support systems (Core Principle 4). There have been growing calls by the UN, for example, during its World Data Forum 2021, for more effective investments from governments, the private sector, civil society, and the philanthropic community to help strengthen data systems; for example, via commitment to the Bern Data Compact for the Decade of Action on the Sustainable Development Goals (https://unstats.un.org/sdgs/hlg/Bern-Data-Compact/). More recently, the *Hangzhou Declaration: Accelerating progress in the implementation of the Cape Town Global Action Plan (CTGAP) for Sustainable Development Data* (https://www.un.org/en/desa/un-world-data-forum-closes-hangzhou-declaration-charting-accelerated-progress-implementation) states that one of the new priorities has been identified as, “Innovation and modernization of national data and statistical systems, with particular focus on addressing the monitoring needs of the 2030 Agenda for Sustainable Development,” as well as, “Dissemination and use of sustainable development data”. Based on these developments, there is potential for governments to pool funds to establish support systems under a credible framework. One such framework is the World Bank-supported Global Data Facility (GDF). The World Bank’s policy briefing has launched two data financing mechanisms, including the UN-hosted Complex Risk Analytics Fund, to support open data ecosystems for crises-related applications, and funding to low- and middle-income countries for investment in data systems^37^. Therefore, there are potential mechanisms and institutions that are being set up to facilitate similar efforts and provide a framework that can be built upon for development of support systems for this community-driven open-science process. Considering that the World Bank announced a campaign to raise $500 million USD over 10 years for data research^37^, it will likely be an attractive prospect for governments and the private sector to fund support systems and to share data samples in return for working solutions that can be implemented locally or duplicated in other regions with minimal effort in the spirit of collaborative development, provided the QA/QC systems are satisfactory.

There are also existing online programs, such as Google Earth Engine, CASEarth Big Earth Data Platform, Global Earth Observation System of Systems, UN Global Pulse, and GDF, that can be used to organize and host support systems needed for the community-driven process^38–41^. Alternatively, initiatives such as Invest in Open Infrastructure (IOI) argue for community-owned open infrastructure to promote research. These initiatives rely on external funding and donors to support specific projects and provide interesting opportunities to identify and organize community-driven DPG projects under international frameworks such as the UN. Despite uncertainty regarding the exact sources of funding, there are encouraging signs that institutions such as the World Bank and the UN are leading new initiatives to attract public and private funding to support development of data infrastructure and resources that can also support the proposed community-driven approach toward DPGs for SDGs.

### Data and model standardization challenges

SDG indicators are quantitative assessments of economic, environmental, and social progress. Developing any DPG related to SDG indicators therefore requires access to relevant datasets, as introduced by Core Principle 4. One issue is that several SDG indicators still lack sufficient data. Additionally, the adoption of a community-supported framework for open science might prevent different statistical agencies from openly sharing data due to national legal restrictions on security, privacy, and commercial laws. For software and model development, this data sharing challenge may be mitigated by developing a set of standardized datasets that are publicly accessible, with the aim of testing and evaluating the performance of new methods, which is a common practice within the data science community. The best performing models and methods using standard metrics and standardized datasets can then be used by NSOs, according to their digital environments, to verify performance for relevant scales. This solution would help achieve Core Principle 2 (see L3 in Fig. 1). Alternatively, encouraging the use of publicly available resources, notably remote sensing data, might help mitigate data access issues and support the development of globally usable multi-scale products.

Several experts highlighted the importance of data and model standardization and the interoperability therein. This is because, traditionally, diverse formats and systems have been used for data collection by a plethora of organizations in multiple countries due to their differences in purpose and utilized instruments. Furthermore, heterogeneity also exists between and among the public and the private sectors. As a result, the data and model frameworks governing the collection and analysis of data related to SDG indicators are characterized by heterogeneous and changing capabilities. All this diversity complicates the standardization and interoperability processes among different regions, especially for community-driven development, as discussed in this study.

Open science and the collaborative development process highlighted in this paper would benefit from the initiation of a UN program to develop a database that collects data samples while preserving the formats and structure of relevant datasets of different countries and systems on SDG indicators. This action could strengthen the work of the developer community and enable the data science community to understand the structure and formats of a wide range of datasets. Furthermore, this would serve to develop programs and processes to create standard datasets, to fill in some data gaps using alternative sources, and to devise interoperability and standardization solutions, while also supporting the objectives of Key Action 5.4.

### Capacity, politics, and acceptability challenges

Several experts underlined the need for capacity building in all countries, and in particular developing countries, starting with landlocked and small island states. The survey results suggest that some regional and societal constraints and technical differences may reduce the ability to develop and adopt DPGs for SDG indicators. The open science approach, integrated with programs for the development of human resources and supported by international organizations and the UN, can contribute to rapidly improving the technological capacity of competent organizations in different countries. This, however, needs to be organized in parallel with digital infrastructure and accessibility development programs, which are considered under the Secretary-General’s Roadmap for Digital Cooperation. More generally, all DPGs approved at L2 (Fig. 1) should be supported by a user-oriented training program for educational and skill development in line with Core Principle 6.

Another major challenge the experts highlighted was the legislative and political obstacles that must be overcome to improve cooperation between countries. This action needs to be addressed by different stakeholders. The Multi-Stakeholder Forum on Science, Technology and Innovation for the SDGs (STI Forum), convened by the United Nations Economic and Social Council (ECOSOC), is an important platform to support this process and to improve accessibility to technology for developing nations to build their capacity toward digital development. Political, social, and governance challenges were also highlighted as part of the grand challenge of building institutional trust around the development of open-source projects. Efforts at the policy level (Fig. 1, L4) can help streamline this process. Additionally, through community-supported open-source projects, it will be possible to recognize and adopt a variety of implementation choices. Pilot studies and demonstration cases by NSOs, based on the proposed solutions, can help inform decision-makers of a scientific consensus using the best implementation methods (Key Action 5.3).

### Way forward

Rapid global progress in science and technology has been the primary driver for unprecedented development, innovation, and progress in human society. These innovations have demonstrated advantages in a variety of societal, economic, cultural, and environmental aspects. In particular, digital technologies and their applications have heavily impacted all levels of socio-economic classes around the world in the past few years.

As such, the experts in the conducted survey agree that DPGs have enormous potential to facilitate global sustainable development. Experts highlighted how big data, artificial intelligence, geospatial data, and the latest digital technology tools are crucial for a wide range of applications related to monitoring and managing natural and human systems. Based on the analyzed responses and suggestions, this report proposes six fundamental principles and several related key actions necessary for the systematic development of DPGs for the generation and maintenance of the SDG indicators.

These core principles are provisional but provide a good starting point for initiating a comprehensive dialogue highlighting DPGs for the SDGs as a concept. Future actions include refinement of the core principles and the development of a consensus to facilitate the key actions introduced. The Summit of the Future and the Digital Compact provide opportunities to discuss these concepts and gather global support for the actions suggested in this manuscript to achieve the 2030 Agenda for Sustainable Development. The propagation and implementation of these principles require a global platform and support from intergovernmental mechanisms.

The best chance to create a reasonable and standardized framework for developing DPGs lies in cooperation between multiple stakeholders and the UN. Actions for the further discussion of possible processes and standards, the collection of existing problems and promising solutions, and the collection of further suggestions and comments from the international community can contribute to this cooperation. It is also necessary to coordinate activities between multinational initiatives focusing on the broad theme of DPGs for the SDGs on the one hand and open science challenges on the other. Once the discussion is finalized and a viable system has been produced, the last hurdle will be political commitment to implement the strategy; this will be essential to harness the full potential that DPGs offer in achieving the SDGs.

The presented analysis offers a preliminary process inspired by expert comments and based on the core principles inferred from the survey responses for an open-source development environment required to build a system of DPGs for SDG indicators. The process aims to inspire innovation by engaging a community of talent that can contribute expertise to the creation of a grassroots movement working toward global sustainability. The process would incorporate the necessary QA/QC measures and policy oversight to ensure multi-stakeholder engagement at national and international levels. The community-driven open-science process proposed is a step toward organizing international efforts to direct research on a larger scale and could be highly valuable as we move forward into the second half of the 2030 Agenda for Sustainable Development. There is great potential in the proposed approach to ensure that digital resources relevant to monitoring SDGs are regularly updated to incorporate rapid developments in science and technology, ensuring that their benefits remain accessible around the world to support information, science-driven policy, and decision support systems.

## Methods

**Fig. 2:**
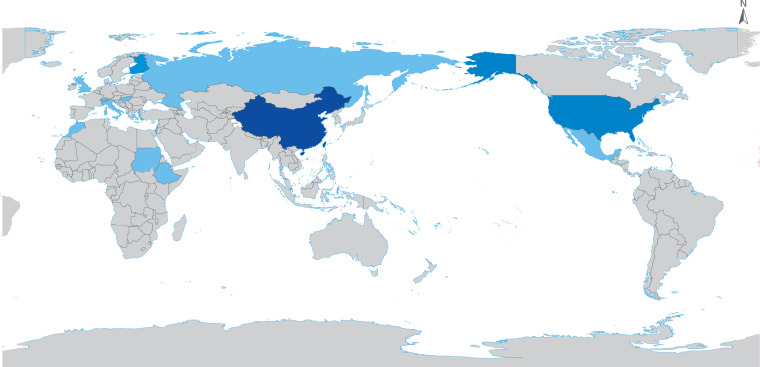
Geographic scope of the survey, showing the number of respondents per country ranging from 1 (light blue) to more than 5 (dark blue).

CBAS conducted a consultative survey with the aim of benefiting from the diverse expertise of a group of international experts. The goal was to shape a broad conceptual framework based on the experiences of a set of relevant experts who have worked in multiple international initiatives, projects, and organizations in the fields of Earth science, Digital Earth, big data, Earth observation, and other related fields. The survey was communicated via email to more than 300 individuals from a database created from the registrants of conferences organized by CBAS over the past five years, without emphasis on concentrating survey results from any particular region. The experts were asked to help identify opportunities and challenges in the conception of DPGs for SDGs, and were requested to share: (a) their opinions and ideas on the viability of DPGs for SDGs; (b) their suggestions on core principles; (c) a set of key actions necessary to develop the proposed framework; (d) a list of existing resources to help realize and drive the process; and (e) the most important challenges expected in the realization of this international framework, as summarized in Section 3. In all, 51 experts from 13 countries/regions responded to the invitation to participate in the survey (Fig. 2). Out of these experts, 36 were from China, including 1 from Chinese Hong Kong. The remaining 15 experts were from various regions around the world. Specifically, there was 1 expert each from Israel, Ethiopia, Russia, Switzerland, Mexico, Morocco, the United Kingdom, Italy, Sudan, and Hungary, with 3 experts from the United States and 2 experts from Finland. The survey design was based on several key points agreed upon by all authors and later approved following reviews by all authors before final circulation. The first key point was that the questions should remain open-ended to allow freedom to respondents to introduce ideas and concepts in their own words to accurately reflect their views. Secondly, a list of keywords was also agreed upon to limit the scope of the responses to a manageable level and ensure that responses meet the necessary context. These keywords included “digital public goods”, “SDGs”, “science, technology, and innovation”, “data and information”, and “multi-stakeholder cooperation”. Thirdly, the survey sought to avoid excessive ambiguity by designing questions that are direct but still consultative in nature.

The survey, which has been provided in this article’s supplementary materials, was sent to respondents with a concept note to share a compiled summary of UN actions and interests toward DPGs and references or links to relevant documentation and websites^4,7,15^. The survey responses were proofread for basic grammar and clarity, though an effort was made to retain original keywords and phrasing. All experts were asked to share their opinions in detail and elaborate on topics coinciding with their personal research directions and interests. The concept notes also stated that the goal of the survey was to collect diversified yet practical opinions from the experts for each question. The experts were also requested to preferably limit their response to a maximum of 500 words to encourage descriptive but concise responses to each question. The survey itself consisted of a total of seven questions. The questions can be divided into four groups, with the first two questions focusing on a broader understanding of the concepts and perspectives of each individual respondent. The second group of questions (3 and 4) was designed to collect core principles and key actions from all respondents. Questions 5 and 6 focused on the likely challenges of solution implementation due to the dynamic changes in the digital landscape. The final question asked the respondents to predict the potential for strengthening multi-stakeholder cooperation concerning DPGs.

The analysis of the responses was conducted manually by CBAS, going through each individual survey response. Following the anonymization of responses, ideas were first collated from all responses received from the experts. The expert opinions were grouped together to define the proposed DPG concept and existing resources were highlighted for developing DPGs for SDG indicators (for the results, see *Results*). A set of core principles was compiled along with relevant key actions, as suggested by the experts (see *Defined core principles and key actions*). Then, through extensive deliberation and exchange of ideas between all authors within the context of the responses received, the preliminary concept for a community-driven approach was outlined and refined. Following feedback from multiple reviews of the subsequent drafts, the proposed finalized approach, policy recommendations, and possible solutions were adopted. The proposed preliminary concept conformed to a set of core principles that were identified by the experts. For a discussion of the identified challenges and a proposed framework building on the ideas and opinions expressed by the experts, see *Discussion*.

### Supplementary information

Survey on a Framework for Digital Public Goods for SDGs

## Data Availability

The anonymized survey responses collected and analyzed during this study are included in the supplementary information files provided with this article (10.6084/m9.figshare.23692758)^42^.

## Code availability

No custom code was written to analyze the survey data described in this article.

## References

[1] BDI. *The Digital Transformation of Industry*. https://www.weforum.org/reports/digital-transformation-of-industries/ (2016).

[2] UN Secretary-General’s High-Level Panel on Digital Cooperation. *The Age of Digital Interdependence*. https://digitallibrary.un.org/record/3865925 (2019).

[3] United Nations. *Report of the Secretary-General Roadmap for Digital Cooperation*. https://www.un.org/en/content/digital-cooperation-roadmap/assets/pdf/Roadmap_for_Digital_Cooperation_EN.pdf (2020).

[4] United Nations. *Our Common Agenda – Report of the Secretary-General*. https://www.un.org/en/content/common-agenda-report/assets/pdf/Common_Agenda_Report_English.pdf (2021).

[5] Cavalcanti DR, Oliveira T, de Oliveira Santini F (2022). Drivers of digital transformation adoption: A weight and meta-analysis. Heliyon.

[6] Guo H, Wang L, Chen F, Liang D (2014). Scientific big data and Digital Earth. Chin. Sci. Bull..

[7] DPGA. *Digital Public Goods Alliance 5 Year Strategy (2021–2026)*. https://digitalpublicgoods.net/DPGA_Strategy_2021-2026.pdf (2021).

[8] OECD. Evaluating policies for delivering agri-environmental public goods. (2012).

[9] Defining Global Public Goods. in *Global Public Goods: International Cooperation in the 21st Century* (eds. *et al*.) 0. 10.1093/0195130529.003.0001 (Oxford University Press, 1999).

[10] Kenny, C. *What are Global Public Goods?* 3–4 http://www.jstor.org/stable/resrep29762.4 (2020).

[11] Long D, Woolley F (2009). Global Public Goods: Critique of a UN Discourse. Glob. Gov..

[12] Bratspies RM (2010). Global Public Goods: An Introduction. Proc. ASIL Annu. Meet..

[13] Guo, H., Goodchild, M. F. & Annoni, A. *Manual of Digital Earth*. 10.1007/978-981-32-9915-3 (Springer, 2020).

[14] Sæbø, J. I., Nicholson, B., Nielsen, P. & Sahay, S. Digital Global Public Goods. *CoRR***abs/2108.09718**, (2021).

[15] UNESCO. *UNESCO Recommendation on Open Science*. https://unesdoc.unesco.org/ark:/48223/pf0000379949 (2021).

[16] Nicholson, B., Nielsen, P., Sæbø, J. I. & Tavares, A. P. Digital Public Goods for Development: A Conspectus and Research Agenda. in *Freedom and Social Inclusion in a Connected World* (eds. Zheng, Y., Abbott, P. & Robles-Flores, J. A.) 455–470 (Springer International Publishing, 2022).

[17] Brunton, S. L. & Kutz, J. N. *Data-Driven Science and Engineering: Machine Learning, Dynamical Systems, and Control*. 10.1017/9781108380690 (Cambridge University Press, 2019).

[18] Guo H (2020). Big Earth Data science: an information framework for a sustainable planet. Int. J. Digit. Earth.

[19] GEO. *Earth Observations in support of the 2030 Agenda for Sustainable Development*. https://www.earthobservations.org/documents/publications/201703_geo_eo_for_2030_agenda.pdf (2017).

[20] United Nations. *Transforming our World: The 2030 Agenda for Sustainable Development*. https://sustainabledevelopment.un.org/content/documents/21252030%20Agenda%20for%20Sustainable%20Development%20web.pdf (2015).

[21] Kulmala M (2021). Atmospheric and ecosystem big data providing key contributions in reaching United Nations’ Sustainable Development Goals. Big Earth Data.

[22] Leonelli, S. *Data-Centric Biology: A Philosophical Study*. (University of Chicago Press, 2016).

[23] Sachs, J., Kroll, C., Lafortune, G., Fuller, G. & Woelm, F. *Sustainable Development Report 2021*. 10.1017/9781009106559 (Cambridge University Press, 2021).

[24] Guo, H. *Big Earth Data in Support of the Sustainable Development Goals (2019)*. (Science Press and EDP Sciences, 2019).

[25] Guo, H. *Big Earth Data in Support of the Sustainable Development Goals (2020): China*. (Science Press and EDP Sciences, 2020).

[26] Guo, H. *Big Earth Data in Support of the Sustainable Development Goals (2020): The Belt and Road*. (Science Press and EDP Sciences, 2020).

[27] Guo, H. *Big Earth Data in Support of the Sustainable Development Goals (2021): China*. (Science Press and EDP Sciences, 2021).

[28] Guo, H. *Big Earth Data in Support of the Sustainable Development Goals (2021): The Belt and Road*. (Science Press and EDP Sciences, 2021).

[29] Guo H, Liang D, Chen F, Shirazi Z (2021). Innovative approaches to the Sustainable Development Goals using Big Earth. Data. Big Earth Data.

[30] Guo H, Chen F, Sun Z, Liu J, Liang D (2021). Big Earth Data: a practice of sustainability science to achieve the Sustainable Development Goals. Sci. Bull..

[31] Guo H (2022). Measuring and evaluating SDG indicators with Big Earth Data. Sci. Bull..

[32] Guo H, Wang L, Liang D (2016). Big Earth Data from space: a new engine for Earth science. Sci. Bull..

[33] IATT. *Emerging science, frontier technologies, and the SDGs - Perspectives from the UN system and science and technology communities*. (2021).

[34] Schroeder, R. Big Data: Shaping Knowledge, Shaping Everyday Life. in *Social Theory after the Internet* 126–148. 10.2307/j.ctt20krxdr.9 (UCL Press, 2018).

[35] Wilkinson MD (2016). The FAIR Guiding Principles for scientific data management and stewardship. Sci. Data.

[36] Nicholson B, Nielsen P, Sahay S, Sæbø JI (2022). Digital public goods platforms for development: The challenge of scaling. Inf. Soc..

[37] United Nations. *Unlocking Data For A Better, Greener, Safer Future*. https://www.data4sdgs.org/sites/default/files/file_uploads/World%20Bank%20Spring%20Meetings%20-%20Unlocking%20Data_0.pdf (2022).

[38] Guo H (2017). Big Earth data: A new frontier in Earth and information sciences. Big Earth Data.

[39] Ji, L., He, G. & Yan, D. Data Sharing in CASEarth Project. *Biodivers. Inf. Sci. Stand.*, (2019).

[40] Guo H (2023). SDGSAT-1: the world’s first scientific satellite for sustainable development goals. Sci. Bull..

[41] Liu J, Wang W, Zhong H (2020). EarthDataMiner: A Cloud-Based Big Earth Data Intelligence Analysis Platform. IOP Conf. Ser. Earth Environ. Sci..

[42] Liang D (2023). Figshare..

