# Supplementary material for: A future for digital public goods for monitoring SDG indicators
Source: Sci Data. 2023 Dec 7;10:875. doi: 10.1038/s41597-023-02803-x (PMC10703768; doi:10.1038/s41597-023-02803-x)
Supplement: Supplementary file 1 — Survey on a Framework for Digital Public Goods for SDGs [file 41597_2023_2803_MOESM1_ESM.docx]

Survey Form

CBAS Expert Survey on a Framework for Digital Public Goods for SDGs

| Respondent Information | |
| --- | --- |
| Title |  |
| Name |  |
| Organization |  |
| Specialization |  |

**Expert Survey Questions**

| **Question 1**\| Do you believe that digital public goods related to SDGs can help to improve global capacity for science, technology and innovation in implementations of the UN 2030 Agenda? |
| --- |

| Respondent # | Response |
| --- | --- |
| 10101 | Yes. Facilitating the discovery, development, use of, and investment in digital public goods, especially in low- and middle-income countries, is expected to accelerate the attainment of the SDGs. |
| 10102 | Yes. |
| 10103 | Yes, undoubtedly an increase in the availability of data, research results, and experience of leading expert groups will make it possible to address the issues of sustainable development at the regional and global level more effectively. |
| 10104 | Yes, I firmly believe that digital public goods are crucial. |
| 10105 | The promotion of digital public goods can enhance global technology and innovation capabilities and help promote the realization of the 2030 Agenda.  Digital public goods are digital products for public welfare by nature or in their effectiveness, including software (platform), data, models, standards, etc. For example, digital heritage (historical building information model) is a type of digital public good, which effectively supports the protection and sustainable development of cultural and natural heritage. |
| 10106 | Yes, I firmly believe in it. Since SDGs are a global issue, the sharing of some global digital resources is helpful for improving global capacity for science, technology, and innovation in implementations of the UN 2030 Agenda. |
| 10107 | Yes. |
| 10108 | Yes. |
| 10109 | Yes. Big data analysis is an important means of global technology and innovation capabilities and requires the support of global data public goods. |
| 10110 | Yes. |
| 10111 | Yes. In particular, they hold the potential to help fair and just access to data, information, knowledge, methodologies and approaches for bridging the digital gap. |
| 10112 | Yes, but I believe that the question is not well formulated. The digital public goods shall improve the global capacity to process Big Earth data, and that should then facilitate the development of technological solutions to support the UN SDGs. |
| 10113 | (Answers were submitted together with respondent 10106) |
| 10114 | Yes. |
| 10115 | Digital public goods can provide the public data, products, methods, and platforms needed to monitor and evaluate global sustainable development goals and promote the realization of sustainable development goals. |
| 10116 | (Advised on the survey’s general design and structure without answering the questions) |
| 10117 | (Answers were submitted together with respondent 10118) |
| 10118 | Yes, they are a very important component for future society. Big data related to environment, society, economics, and politics can especially bring useful knowledge for humankind. |
| 10119 | Yes, they can play a large part. |
| 10120 | The sharing of Earth observation data, algorithms, products, analysis models, etc. is conducive to the development of related scientific applications, so that the potential of data applications can be fully realized. Feedback of the applications to the data itself is also conducive to the development of sensors, satellite platforms, etc. Therefore, digital public goods can help enhance global technology and innovation capabilities. |
| 10121 | Yes, digital public goods will play a huge role in supporting core social functions and infrastructure. |
| 10122 | Yes. It helps to enhance global technology and innovation capabilities. For example, the extensive use of the series of ground data shared by the European Space Agency (ESA), such as Sentinel, does have a greater role in promoting technology and innovation. |
| 10123 | Yes. Digital public goods provide the sharing of data and information, help to enhance global technology and innovation capabilities, and promote the realization of the 2030 Agenda. |
| 10124 | Yes. The promotion of digital public goods can enhance global technology and innovation capabilities, thereby promoting the realization of the 2030 Agenda.  Digital public goods are digital and informatized data products launched by enterprises, social organizations, and groups that use information technology to fulfil their social responsibilities and promote the realization of SDGs. |
| 10125 | They will help enhance regional and global technology and innovation capabilities and promote the realization of the 2030 Agenda. |
| 10126 | Yes. Digital public goods can allow people in different regions and industries around the world to use the data and mine data/information from different perspectives, which is conducive to promoting multiple innovations and helping the realization of the 2030 Agenda. |
| 10127 | Yes. |
| 10128 | The promotion of digital public goods will help the world achieve sustainable development goals, especially digital public goods for specific fields such as agriculture, heritage, and climate change, which can effectively bridge the digital divide. |
| 10129 | I strongly believe they will, because the UN 2030 agenda spans across the entire spectrum of human society. Informed decisions and policies are the cornerstone of prosperity at all levels—individual, corporate, or national. How effective that turns out to be depends much on levels of access to data, tools, and skills, which is what open-source development and interdisciplinarity would potentially deliver. |
| 10130 | Yes. |
| 10131 | Yes, digital public goods can help collect, analyze, and communicate key information on successful SDG achievements, and challenges to achieving SDGs. Well-designed digital public goods can allow for better management of data, leading to better collaboration, which eventually can lead to the right information communicated to the right people for better decision making. SDGs cover dynamic topics that need information on developing realities in a changing world in order to address the UN SDGs.  Spatial distribution of factors relating to SDGs such as population, services, environmental change, land use, etc. are important to understand and respond to the dynamics of SDGs. |
| 10132 | Yes. |
| 10133 | According to the DPGA definition, digital public goods need to have "open-source software, open data, open artificial intelligence models, open standards and open content, comply with privacy and other applicable international and domestic laws, be industry standards or best practices, and not cause damage". Just as a series of open-source software and open-source communities (such as GitHub) have contributed to the research & development field, the open-source attributes and open standards of digital public goods will greatly enhance the technology and innovation capabilities, and promote the realization of the 2030 Agenda. Digital public goods can be platforms, software and hardware devices, digital twin communities, etc. that follow open-source protocols. |
| 10134 | Yes. Global technology and innovation itself cannot be separated from the current digital Internet technology. It would be even better if existing practices could be effectively abstracted into digital public goods. Because of the characteristics of digital public goods—open content, open standards, open data, etc., all of which involve intellectual property rights and user privacy protection—there are still many difficulties. |
| 10135 | Yes. The UN 2030 Agenda cannot be met unless there are further advancements in science, technology, and innovation. |
| 10136 | Yes. Digital public goods can and will help the implementation of the UN 2030 Agenda globally, regionally, and locally, including in developing countries (low- and middle-income countries).  However, this ability to help is not unconditional and it might not happen at any rate.  The digital public goods are understood as open goods (a) serving for common use, (b) created by very different stakeholders (governmental, private, civil, individuals) and (c) being characterized with diversified properties (different nature, specifications, purposes, quality, attributes etc.). Concepts, initiatives, experiences, organizations, cooperation, assistance, and faith are necessary for their identification, access, assessment, transformation to common standards, and dissemination for SDG purposes, etc. The political wishes, finances, forces and support, selective projects, and targeted platforms are of high importance on the way to deployment of digital public goods for successful implementation of the SDGs. Commitment of the stakeholders, solidarity, science, technology, exceptional endeavours, efforts, and well-managed cooperation are the means for our common contribution to “not missing the last chance”.  As for how to realize the promoting role of digital public goods, a partial solution is available in the Reuse of Public Sector Information (PSI) Directive of the European Union (Directive 2003/98/EC) entered into force on 31 December 2003. |
| 10137 | Yes. |
| 10138 | Yes, but only if they are acted on. |
| 10139 | Yes, I do think so. |
| 10140 | Digital public goods help to enhance global technology and innovation capabilities and promote the realization of the 2030 Agenda! |
| 10141 | Digital public goods can help enhance global technology and innovation capabilities, and then promote the realization of the 2030 Agenda. |
| 10142 | The promotion of digital public goods can indeed enhance global technology and innovation capabilities, but I don't know much about the 2030 Agenda, and I haven't found relevant content.  Digital public goods should refer to open and shared, free and open-source digital content, such as: open-source software, open data, open artificial intelligence models, open standards, and open content. |
| 10143 | As a derivative product of the digital economy and modern science and technology, digital public goods can help realize the 2030 Agenda. |
| 10144 | Yes. |
| 10145 | Yes. The emergence of digital public goods, especially those related to human activities and the general public, will stimulate public enthusiasm and feedback on scientific research. Researchers can also extract innovation points from this positive feedback, and then promote the realization of the 2030 Agenda. |
| 10146 | Yes. |
| 10147 | Digital public goods can enhance global technology and innovation capabilities and have practical significance in realizing the 2030 Agenda. Digital public goods monitor and evaluate the 2030 Sustainable Development Goals in the form of data, products, algorithms/models, digital platforms, etc. They provide decision support reports for the formulation and application of sustainable development policies, advocate public behavior, and provide digital public goods to support the 2030 Agenda. |
| 10148 | Yes, I believe that the digital public goods of SDGs can help to improve the global capacity in implications of the UN 2030 Agenda. |
| 10149 | Yes. |
| 10150 | Digital public goods are able to help scientific innovation and therefore the UN 2030 Agenda. |
| 10151 | They can contribute to enhancing global science and technology and innovation capabilities, thus promoting the realization of the 2030 Agenda. |

| **Question 2**\| What are some key digital resources and technologies required as digital public goods to facilitate rapid implementation of SDGs globally? |
| --- |

| Respondent # | Response |
| --- | --- |
| 10101 | Some examples could be electronic tools, systems, devices, and resources that generate, store or process data. This includes information and communication technologies (including smooth radio-communication services), ICT infrastructure, networks, and services, etc. |
| 10102 | Some ideas are global scale SDG products, especially on human habitats with indicators like NOx, SOX, PM1.5 and PM10 etc. |
| 10103 | Within the geosciences, priority is given to open standardized data, or data with high-quality metadata, regularly received from Earth remote sensing satellites by various national agencies, as well as data from ground-based observation stations in the fields of meteorology, seismology, oceanology, socio-economic development of territories, etc.  The results of environmental observation data processing, obtained, among others, as a result of modelling the development of processes on land, in the atmosphere and ocean using the principles of deep data learning, can also be considered as key information resources, especially at the level of decision-making and territory management.    Data models and open-source software for processing and analysing open data will allow scalable solutions for various SDG targets.  Public open data, which is generated by users in open services such as Wikimapia or weather station holders, is also a key information resource, especially where there is no standardized open data network. |
| 10104 | In my field of disaster and humanitarian medicine and public health, open access databases and knowledge hubs will facilitate rapid implementation of SDGs globally. |
| 10105 | Big Earth data in digital form would help (data in the economic, social, and environmental fields). |
| 10106 | Strengthening the effective fusion of multi-disciplinary data resources such as global remote sensing, meteorology, and agronomy, and the application of spatiotemporal big data processing technologies such as artificial intelligence are the keys to facilitating rapid implementation of SDGs globally. |
| 10107 | I personally think that public data products based on space observations should be one of them. |
| 10108 | First, datasets. A digital ecological environment database and a digital model of disaster prevention, mitigation, and early warning could prevent disasters caused by extreme climate.  Second, digital public platform. This might include open-source software, retrieval platforms, and standard content that provide information channels for the public to obtain digital public goods, and also provide an open platform where everyone can contribute data and innovation to promote sustainable development.  Third, a central system for the effective coordination of systems and platforms. |
| 10109 | I would say data and products on a global scale, as well as shared digital technology implementation methods. |
| 10110 | Some ideas are interoperability, (spatial) data infrastructure, a data cube, artificial intelligence, and high performance computing. |
| 10111 | Mainly, in addition to satellite remote sensing data, regular updates of information on natural resources and environment, such as air, land, soil, water, biodiversity, and ecosystems, are still key digital resources to be addressed. Concerning technologies, it is essential for developing countries to acquire knowledge and capacity, and in particular, to strengthen multisource data acquisition, analytical tools and models, as well as capacity development for combined analysis of spatial data with social and economic data. |
| 10112 | What is mostly required is services to process Big Earth Data. Since we do not have a common set of services, each researcher is developing their own set and this produces duplication, loss of time and loss of resources.  While if the Centre for Big Data in support of the UN SDGs can, with our assistance, develop a series of associated services, that will significantly assist the international community. |
| 10113 | (Answers submitted together with respondent 10106) |
| 10114 | Long-term global natural environment and socio-economic digital public goods with good consistency and dynamic updates would aid SDG implementation. |
| 10115 | Common and easily accessible data, products, methods, and platforms are essential tools. |
| 10116 | N/A |
| 10117 | (Answers submitted together with respondent 10118) |
| 10118 | I would like to emphasize my own expertise. We need timely and accurate data about our common environment and one good example for technology is SMEAR (Station for Measuring Ecosystem-Atmosphere Relations) that I have used and developed extensively during the last 35 years. Big data from SMEAR can be a base for smart technologies related to atmospheric and air quality issues. |
| 10119 | The main necessary resources are essentially financial. |
| 10120 | Shared data, open-source software or algorithms, shared products, open-source artificial intelligence models or related professional models, and related standards and norms can all rapidly promote global sustainable development. |
| 10121 | Open-source software, open datasets and data sharing platforms, such as digital systems and applications that serve specific populations with specific SDG goals, will help implementation. |
| 10122 | Open-source software, shared data, open-source artificial intelligence models or related professional models, and related standards and norms can all rapidly advance global sustainable development. |
| 10123 | We could use open-source software, open data, and open AI models. |
| 10124 | Digital public goods that can rapidly promote global sustainable development include: various observation data, socioeconomic statistics, spatial basic geographic data, and model calculation data. |
| 10125 | Data sharing, data governance, and AI analysis are all essential to SDG achievement. |
| 10126 | Big Earth data (remote sensing, basic geographic information data, geographic survey data) and social and economic survey data related to SDG indicators can be used as digital public goods to rapidly promote global sustainable development. |
| 10127 | Digital Farmland and digital agriculture are two examples. |
| 10128 | The key digital public goods should be various original basic data products used to construct SDG indicators, such as traditional statistical data (such as demographic data, economic data, etc.); thematic data of objects (forest, cultivated land and wetland, etc.), inversion of surface parameter data (surface temperature, surface humidity, surface deformation, etc.); other basic geographic data (such as traffic road network, traffic flow, etc.); disaster prevention and reduction, global grain production distribution, per capita consumption data, etc. |
| 10129 | The digital technologies and resources for public goods are known to facilitate the 2030 Agenda—they, typically, revolve around financing on the one hand and data sharing and modelling infrastructure on the other. However, both sides are constrained by spatiotemporal variations in the form of legislation, geo-political and socio-economic and cultural variations. The impact of these variations is more pronounced in some countries than in others, with the developing world being disproportionately affected. The non-orthogonality of SDGs entails coordinated efforts across these barriers, thus, while we may identify the resources and technologies, practical unification of efforts across geographical borders remains the most lacking initiative. |
| 10130 | Analytical models and tools and continuous data streams providing natural and social observations would all contribute to SDGs. |
| 10131 | Open mapping tools such as spatially enabled mobile data collection tools allow for real world data and verification of remotely sensed data to capture and update the detailed reality of a situation. Open Geographic Information Systems (QGIS) analyze spatial data for patterns to better understand complex topics. Open interactive spatial data analysis dashboards and QGIS visualization tools communicate patterns to better understand dynamic landscapes of SDG topics. |
| 10132 | Existing tools and techniques require knowledge sharing for successful implementation. |
| 10133 | Large-scale open-source community, open digital centralized platform, open data aggregation platform, open-source visual rendering engine, and a geospatial engine are all ideas to help SDGs. |
| 10134 | It is a good idea to build an open digital ecosystem database as a digital public good. However, it is more difficult to operate it because many countries consider environmental data to be private and security data and are unwilling to disclose it. Therefore, it is recommended to fully disclose the metadata of the data. If this is done, it will allow everyone to use these open data, but at the same time protect the privacy of the data provider to a certain extent. |
| 10135 | Key digital resources are:   - Big Data (Science is now Big Data centric) - Global Open Science Cloud (GOSC), which is based on open science and open data. The digital infrastructure in GOSC is peer-to-peer federated, which means that big data can now be processed at any location due to the FAIR Principles (findable, accessible, interoperable, and reusable) being implemented globally. - SDG Platform   - The application software for processing the SDG datasets should be open to innovation.   - The data collection and metadata should reflect this software innovation. |
| 10136 | I give my ideas below.  Almost every sustainable development goal, with the respective targets and indicators, has location-based characteristics. Therefore, the EO and GI data with their respective software, models, standards, and technologies are key digital resources and technologies for rapid implementation of SDGs, globally, regionally, and locally.  Hereby, I suggest having Earth science digital public goods or Big Earth Data (that is the information generated on air, water, ecosystems, and biodiversity etc.) interpreted as (a) widened to EO and GI, and (b) expanded by listing the topics of land ownership, land tenure, and land use having an emphasized role among the SDGs.  The EO and GI information are inevitable references (a) in the process of implementation of SDGs and (b) in the course of reporting, identifying and decision-making about fulfilment or non-fulfilment of an indicator/target/goal. The functions of EO/GI information are to:   - record status or phase of phenomena, - monitor changes in status or phase of the phenomena, - ensure transparency of the phenomena surroundings, - be demonstrative and evidence-based, - be attributable and manageable for computer methods, - provide authentic documentation in a conventional way, - provide factual means and resources in environmental, economic, and social reports and evaluations that are substantial bases in professional and political decision-making both (a) over geographical boundaries (in- and across-countries, local, regional, and global) and (b) between different sectors. |
| 10137 | SDGs need a clear definition of the indicators, space observation, freely accessed remote sensing data, etc. |
| 10138 | Data focused on discrete SDG issues/problems that meet FAIR guidelines, open-source software that manage relevant databases and datasets, open-access research articles that are peer-reviewed and focused on SDG problem-solving, national and international SDG-focused public remote-sensing satellites and public open science infrastructures are some possibilities. |
| 10139 | Digital technologies, including data acquisition and processing technologies from Earth observations, remote sensing, GIS, GNSS, citizen science and social media, are important for implementing SDGs. |
| 10140 | Data, products, results mapping, suggestions, reports and other digital public goods can rapidly promote global sustainable development! |
| 10141 | Large-scale spatial Earth observation data and artificial intelligence models would all contribute to SDG research. |
| 10142 | Any digital public goods that respect intellectual property rights and user privacy will contribute to promoting global sustainable development. Digital public goods that can rapidly promote global sustainable development, including open scientific data, open artificial intelligence models, open key technologies and data that are conducive to the universal development of society, etc. |
| 10143 | Multi-source digital products that reflect progress towards the Sustainable Development Goals are necessary. |
| 10144 | Global-scale SDG target products, products related to SDG14 including global ocean chlorophyll a concentration, global ocean primary productivity, global ocean acidification products, and global ocean dissolved oxygen products are a few priorities in my field.  Additionally, common key technologies for the development and analysis of SDG target products could be product development technology, data sharing technology, geographical space-time analysis technology, etc. |
| 10145 | A few examples are weather-related digital public goods, disaster-related digital public goods, etc. |
| 10146 | Some ideas are various observation data, statistical data, high-precision and high-performance algorithm models, various information products, big data sharing platforms, cloud computing platforms, etc. |
| 10147 | Digital public goods that can rapidly promote global sustainable development include global or regional assessment reports for the 2030 goals, data products, standardized and open assessment algorithms/models that can be provided free of charge, and digital platforms that can be quickly customized. |
| 10148 | Satellite remote sensing, cloud computing, and artificial intelligence are a few ideas that could help. |
| 10149 | More accurate digital public goods for long-term global environmental elements should be a priority in establishing digital public goods as tools for SDGs. |
| 10150 | Public goods that reflect the process of achieving the sustainable development goals, such as SDG1, other digital public goods such as film and television, image or data sharing platforms, and processes and experience describing poverty eradication in the world or some regions will help implementation of SDGs. |
| 10151 | Open-source software, shared data, open-source AI models or relevant professional models, standards, and specifications are just a couple to name. |

| **Question 3** \| What are the core principles that any framework on digital public goods for SDGs should ensure? |
| --- |

| Respondent # | Response |
| --- | --- |
| 10101 | Provide without the motive of profit to all members of a society, either by government or a private individual or organisation which is to the benefit or well-being of the public. Leave no one behind and do no harm. |
| 10102 | Digital public goods should be equal and mutually beneficial, and open and free. |
| 10103 | Open data is publicly available without restrictions. Technological solutions should provide the possibility of reprogramming, modularization, and recombination to build new open solutions at various spatial and socioeconomic levels. |
| 10104 | The core principles actually overlap with those of humanitarian medicine, including impartiality, neutrality, independence, universality, availability, accessibility, acceptability, and quality. |
| 10105 | Below are a few ideal principles to keep in mind when drafting any digital public good framework:   - Public welfare service. - Broad interest. - Information sharing. - Data security. - Product authenticity. - Voluntary access. |
| 10106 | All frameworks on digital public goods for SDGs should ensure the core principles of commonality, fairness, full open sharing, and sustainability. |
| 10107 | Cooperation and sharing for mutually beneficial targets are essential. |
| 10108 | First, priority should be put on digital public infrastructure.  Second, the principles of human rights, privacy protection, convenience, and security.  Third, the principle of combining universal characteristics and local diversities. |
| 10109 | Any framework should focus on intellectual property, shared norms, standardization of implementation. |
| 10110 | Some of the important principles are openness, neutrality, FAIR compliance, and reproducibility. |
| 10111 | Data standards and regulations as well as institutional arrangements for open data sharing should be considered. |
| 10112 | - Open access. This means accessible to everybody willing to use them. - Based on open access software. This means that the end-user will not be forced to buy a specific software package in order to be able to run and use the digital public goods. - User-friendly. This means that clear instructions will also need to be developed so that any end-user can really benefit from the digital public goods. - Maintained and updated continuously. Software and hardware are rapidly changing. If the digital public good can only run on a specific operating system, when such an operating system does not exist anymore, then the digital public good becomes obsolete and cannot be used. Therefore, the digital public goods will be required to be maintained continuously. - A support help desk will be required in order to guide the end-users when they do have some questions. |
| 10113 | (Answers submitted together with respondent 10106) |
| 10114 | They should include openness, cooperation and sharing. |
| 10115 | Openness, sharing, versatility, availability and accessibility, ease of use, scalability, and security are all key points to be upheld. |
| 10116 | N/A |
| 10117 | (Answers submitted together with respondent 10118) |
| 10118 | Continuous and comprehensive measurement activity. Big data should include these two components no matter what it measures or collects. |
| 10119 | The framework should facilitate access to data and tools. |
| 10120 | It should ensure security, protect privacy, comply with standards and norms, protect intellectual property rights, etc. |
| 10121 | We need to ensure data security, ease of use, and product sustainability. |
| 10122 | It needs to ensure security, protect privacy, comply with standards and norms, protect intellectual property rights, etc. |
| 10123 | Open sharing, data availability, and high credibility are a few examples of principles. |
| 10124 | The core principles that need to be considered in the establishment of the framework mechanism of digital public goods include: the data of digital public goods can be disclosed, the data is real, and the data is traceable. |
| 10125 | Any framework must ensure security, protect privacy, comply with standards and norms, protect intellectual property rights, etc. |
| 10126 | It needs unified raw data, unified data processing standards, and unified analysis methods. |
| 10127 | Digital public goods should be accessible free of charge. |
| 10128 | The core principles of digital public goods should include seeking truth from facts, sharing and equal respect, people-oriented, and respecting intellectual property rights; accessibility, data accuracy, reliable data sources, establishing a mature and feasible data sharing mechanism, and establishing a sound global classification of a digital public goods system. Seeking truth from facts refers to the reliability and scientificity of the data, which is the actual result of the model and method under certain conditions. The data allows errors due to the defects of the method, which can be corrected with the advancement of technology and the improvement of methods, not because of artificial "correction" due to other reasons; the focus of sharing refers to the contribution of the method. With the support of basic data, everyone can use the shared method to obtain consistent results in the area of interest; equal respect should refer to mutual respect for each other's achievements; discuss methods of data improvement together, and help each other with shared support, instead of imposing our own results and opinions on each other. |
| 10129 | For me, three key words stand out: open science, interdisciplinarity and funding. In the last few years my research has focused on Big Data Modelling of SDGs, seeking to uncover triggers of targets from attributes that span across the 17 SDGs. Countries across the world embark upon the SDG project with, typically, country-specific objectives. Practical implementation of any framework should follow the principles of:   - Universality of science, ensuring open data access - Collaboration (as part of 1) - Bindingness (ensuring a degree of obligation in meeting commitments)   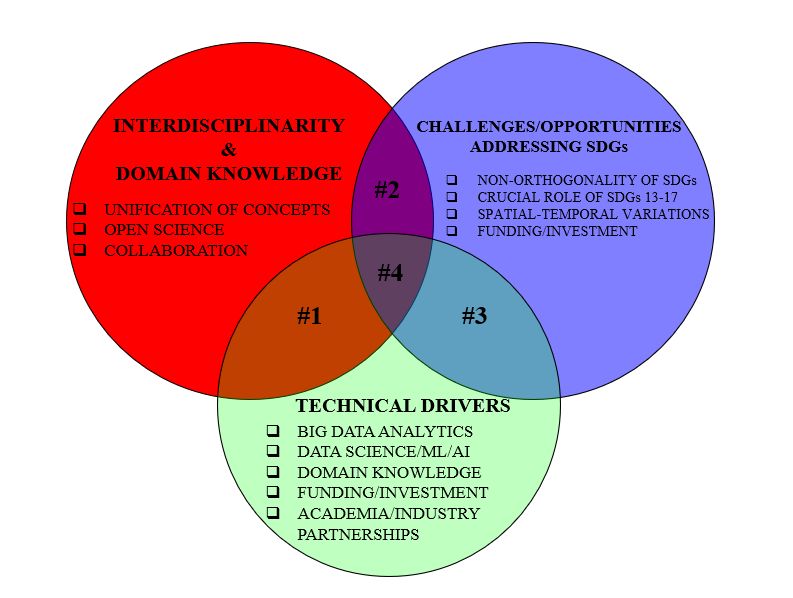 |
| 10130 | Fairness, transparency, interoperability, and security should be included. |
| 10131 | Accounting for different types of data, including tabular, spatial, raster, etc. Also, having different sources and sizes of datasets is critical—some SDGs can be tracked with little data obtained remotely. Other SDGs will require more detailed and larger datasets that will require direct on the ground surveys that need to be updated continuously.  Openness of data. It is important to be able to share resources, and that includes access and sharing of core datasets that multiple SDGs and organizations can use. |
| 10132 | A balanced and flexible market structure is key. |
| 10133 | Quantified contribution and return, open-source convention, and a value certification system are all important principles. |
| 10134 | Data providers should be properly acknowledged and must be cited. |
| 10135 | The core principles are being global and being peer to peer. UN SDGs are interdisciplinary, cross domain, and cross geographic boundaries. |
| 10136 | Based on the concept of “Our Common Agenda” and “CBAS Expert Survey on a Framework for Digital Public Goods for SDGs” I conceive the core principles as follows:   - To create a multi-stakeholder technology track (involving all stakeholders: governments, the UN system, the private sector, civil society, and individuals, including youths) to speed up the SDGs’ implementation. - To utilize science, data, technology, and innovation to facilitate global progress towards sustainable development. - To set multinational initiatives focusing on the broad topic of Earth Observation and Geoinformation (EO&GI) digital public goods for the SDGs, and on the related challenges of open science. - To achieve the goals, a framework must operate as a global project working towards improving recognition, visibility, and accessibility of Digital Public Goods for the SDGs to improve global evaluation and assessment of SDG indicators.   Two special amendments of mine are given below:   - To handle the difficult question “who pays the bill?” I understand that the UN Agenda 2030 serves as an agreement (social? or institutionalized? or governmental?) to answer the above question. However, there are actual expenses when implementing the SDGs or operating any framework. Handling the reimbursement of the expenses must be part of the core principles of any framework. - The core principles should consider global, regional, and local interests, including developing countries, as well. |
| 10137 | Core principles include being accurate, timely, and globally accessible. |
| 10138 | There are many principles that have been articulated for digital public goods, too many to list here. Moreover, there are some principles that are common to all types of digital public goods and some that are specific to data, software, and scientific literature. Also, the authors of some principles are national or international, governmental or non-governmental, top-down or bottom-up, discipline-specific or related to individual SDGs. These should be compiled and evaluated for broad or specific application. |
| 10139 | Open, free, and secure digital future for all must be included in a framework. |
| 10140 | The core principles that need to be observed in building a framework mechanism for digital public goods are: usability, sharing, and sustainability! |
| 10141 | The data should be open and transparent, and the data should be unified and standardized. |
| 10142 | - The principles of fairness, friendship, and mutual benefit: safeguard the interests of both users and data providers. - Privacy protection principles: including that user privacy data will not be shared and used, and personal information must not be tampered with or used. - Integrity principle: content identified as digital public goods should be complete and reusable. |
| 10143 | The framework should focus on public welfare, high quality, high credibility, and being tested for applicability. |
| 10144 | Some of the key principles include:   - Principle of equality. - Principle of equal opportunity. - Principle of multi-stakeholder participation. |
| 10145 | The potential framework should be compliant, transparent, and concise. |
| 10146 | The framework should work on a mutually recognized legal basis, use unified data, and apply universal technical standards. |
| 10147 | The framework for digital public goods needs to consider the principles of quality control, standardization, security, compatibility, ease of use, openness, and availability during the construction process. |
| 10148 | The principles should ensure DPGs are freely and publicly accessible to global communities. |
| 10149 | We should promote open access to ensure equal access to digital public goods for all. |
| 10150 | Ensuring information data rights and security are two important principles. |
| 10151 | We need to think about how to guarantee security, protect privacy, and comply with standards and specifications. |

| **Question 4** \| What are the key actions necessary to ensure digital public goods for SDGs that can lead to improving global access to data and information resources? |
| --- |

| Respondent # | Response |
| --- | --- |
| 10101 | - Regulation and policy have important roles in facilitating the deployment of next-generation infrastructure, harmonizing privacy and data protection rules, and ensuring a level playing field. - Focus attention on mobile infrastructure to allow more marginalized people to access finance and participate in the economy. - Focus should be put on infrastructure, pricing, and skills development to unleash the potential of entrepreneurs on the ground in developing economies. - Unleash the power of partnerships. |
| 10102 | We need to enhance convenience of data collection. |
| 10103 | Some ideas include raising public awareness through popular Internet and mobile services, use of social media, development of intuitive interfaces for accessing data, open and visual recommendations for the use of digital public goods, and teaching digital public goods skills in schools and universities. |
| 10104 | More resources allocated for the less developing countries and local community engagement are key to ensure digital public goods for SDGs. |
| 10105 | - Establish a global protocol for digital public goods. - Construct a classification system for SDG digital public goods. - Implement the Digital Compact by involving all stakeholders. |
| 10106 | Take actions that will enhance international data transfer and sharing, cloud transformation and interoperability, and ensure fairness in the digital environment. This could enable SDG digital public goods to improve global access to data and information resources. |
| 10107 | Carry out data and information cooperation and exchange aimed at promoting the realization of SDGs. |
| 10108 | Create a global digital normative system to achieve efficient data sharing, utilization, and supervision. |
| 10109 | Break through the barriers to global sharing mechanisms and enhance the demonstration effect. |
| 10110 | Identify national/regional leaders that can play the role of creating positive dynamics in their respective areas. Build trust and provide access to only good quality/accountable data and information, then improve education of the younger generations. |
| 10111 | Information application infrastructure and networking of database providers would help access to data globally. |
| 10112 | We must convince the international community that the digital public goods that we will develop are useful, beneficial, and that they will be supported over a certain period of years. This means that researchers will then be willing to adopt such digital public goods, since they know the goods will be available for a long period of time. In other words, the sustainability of digital public goods must be ensured. |
| 10113 | (Answers submitted together with respondent 10106) |
| 10114 | (1) Platform construction: promote the development of digital public goods in accordance with SDGs and build a digital public goods service platform for SDGs. (2) Strengthen scientific research: carry out research related to SDGs supported by digital public goods, and set up related funds and talent funding plans. (3) Develop related applications: focus on services for SDG digital public goods. SDGs support the development of digital public goods to carry out demonstration applications. |
| 10115 | Some ideas are: sharing of digital public goods; establishment of a good and stable global cooperation framework and mechanism; construction of a public platform for people in different groups and fields to facilitate more people's participation and wider cooperation. |
| 10116 | N/A |
| 10117 | (Answers submitted together with respondent 10118) |
| 10118 | There should be open data policies and open sharing of measured data and code used. This will facilitate a wider base for collaboration between sectors. |
| 10119 | Coordination of actions and pooling of resources between different actors are necessary actions. |
| 10120 | Identify digital public goods related to SDG work, and promote the combination of the two through conferences, training, and open discussions. |
| 10121 | Governments of various countries cooperate with relevant institutions and companies. For example, it is possible to solicit optimal digital public goods solutions in a specific area of the SDGs. Additionally, developed countries could guide underdeveloped regions to use digital public goods. |
| 10122 | Find out the gap between digital public goods and SDG work and establish corresponding mechanisms and frameworks to connect them. |
| 10123 | Establish a communication and information service sharing platform. |
| 10124 | Facing the SDGs, CBAS’ work is arranged in a targeted manner, and SDG satellites are used for the production of digital public goods. |
| 10125 | Identify the common ground between digital public goods and SDGs, and use it as a bridge. |
| 10126 | Data sharing can promote data exchange, use, information mining, and play a role in promoting data improvement. Through data sharing, the combination of digital public goods and SDG work can be promoted along with the circulation of global data and information resources. |
| 10127 | We should focus on application and demonstration. |
| 10128 | There are a few ideas for key actions, including: building simple sharing platforms and tools; data resource sharing; data production standardization; publicity, promotion, and especially training for digital public goods are of great significance for promoting the circulation of global data and information resources. |
| 10129 | - Create global SMART objectives, aligning with national goals already set. - Form and agree to integrated strategies, based on unified concepts. - Promote change management at national levels via UN institutions. - Promote interdisciplinary Knowledge Transfer Partnerships and collaboration. - Enhance training and upskilling across sectors/nations. |
| 10130 | New data governance styles, geoscience digital ecosystems, and interoperability standards and definitions could be implemented. |
| 10131 | - Spatial data literacy. There is always a margin of error when it comes to statistics, and categorizing numerical values and findings on maps is no different. Understanding spatial patterns, certainty, and what can be mapped is necessary to utilize digital public goods for mapping. - User friendly open-source technology - Open geographic datasets that have comprehensive world coverage of major SDG-related topics—landcover. - A go-to repository for people working in this space to share and access this data, such as OpenStreetMap. |
| 10132 | Coordination at two levels: orchestrating software development in general; and increased efforts for specific domains and goals, for example: VODAN for COVID. |
| 10133 | Vigorously promote the output of SDG work, build a platform for borderless exchanges and summarization, and deepen cross-field cooperation to increase the depth of the platform. Get more decision support. |
| 10134 | In non-commercial trading activities, some people are willing to share some idle items, land, etc. for exchange. Maybe such a model can facilitate the circulation of global data and information resources? |
| 10135 | For the UN SDGs, ITU is the UN agency involved with digital infrastructure. However, ITU is not aware and not fully engaged with GOSC.  Since AU and LAS follow closely the activities of ITU, ITU must be involved in GOSC. |
| 10136 | I fully support the key actions discussed explicitly or implicitly in the document “CBAS Expert Survey on Framework…”. For the sake of emphasis, I give them as listed below:   - Utilizing science, data, technology, and innovation to facilitate global progress towards sustainable development. - Establishing big data ecosystems with a particular focus on Big Earth Data. - Providing services essential for addressing the most challenging problems facing sustainable development, such as lack of data and technological barriers to implementing the SDGs. - Elaborating and involving methods like data sharing, technological solutions, decision-making support, as well as capacity building for developing countries (but not only). - Developing data infrastructure and information/data products. - Creating new knowledge for SDG monitoring and evaluation. - Developing and launching a series of SDG satellites. - Providing policy and advisory services through an SDG think tank. - Improving global capacity for SDGs in developing countries. - Developing a mechanism to identify, evaluate and standardize new and existing digital public goods relevant to the SDG indicator framework.   However, I suggest three further key actions to be elaborated and solved in the CBAS-planned framework, namely:   - Identifying and accessing the stakeholders (providing the goods, from one side and applying the goods for SDGs, from the other side). - Attaining and managing multi-stakeholder agreements about the digital public goods. - Involving crowdsourced individual stakeholders for utilization of their digital public goods. |
| 10137 | Free data and techniques to the public should be made available. |
| 10138 | SDGs are subsets, albeit very important ones, of larger public issues and activities that focus on access to data and information resources. International cooperation and coordination of all facets is key, but organizations and groups already exist that should not be ignored or duplicated. These should be identified (if they have not yet) and the SDG angle should be established as an express subset. The other approach is to create new ones that are only focused on SDGs, but that cooperate and coordinate closely with the relevant broader international entities. |
| 10139 | Jointly work together with all international organisations and institutions on digital cooperation for achieving and implementing SDGs. |
| 10140 | Establish a shared platform to promote the digital public goods and services for SDGs in the form of projects, conferences, exchange visits, websites, etc., and promote the circulation of global data and information resources! |
| 10141 | Share and publish Big Earth Data products related to SDGs. |
| 10142 | Strengthen publicity and promotion, let more scientific and technological workers and data resource owners understand the goals and functions of SDGs, and let more like-minded people join in this work. Further expand the influence and attract more digital public goods to open and share.  Establish a global digital public goods sharing platform, and global users can apply for use, breaking the barriers to the circulation of global data and information resources. |
| 10143 | Digital public goods should be defined around the SDGs to improve access. |
| 10144 | Open collaborative projects.  Develop data and technology sharing mechanisms. |
| 10145 | Cooperate with leading Internet companies (such as AutoNavi Maps and Baidu Maps) to develop functions that can be embedded in their products, distribute digital public goods based on the Internet platform, and obtain user feedback. |
| 10146 | Strengthen the publicity of digital public goods and SDGs, carry out case studies of digital public goods supporting the realization of SDGs, and form a group of digital public goods with global influence to achieve major achievements in SDGs. |
| 10147 | Through the establishment of an open sharing mechanism, with the help of cloud computing and cloud platforms, demonstrate applications of digital public goods in SDG working areas, release the SDG application cases as digital public goods, do a good job of publicizing related achievements, and establish close links with world-renowned data publishing websites or platforms, thus driving the circulation of global data and information resources. |
| 10148 | Data needs to be made freely and publicly accessible to global communities. |
| 10149 | Create an interdependent system between digital public goods and SDGs, and ensure smooth communication between decision-makers, scientists, enterprises, and civil society actors. |
| 10150 | Create an SDG data sharing platform for government departments and academic institutions to carry out research, so as to promote the circulation of global data and information resources. |
| 10151 | Find the differences between digital public goods and SDG work and establish corresponding connections. |

| **Question 5** \| What are the likely challenges in adopting the approach to implementing digital public goods for SDGs? |
| --- |

| Respondent # | Response |
| --- | --- |
| 10101 | A few potential challenges include:   - Stable and long-term financing. - Data security. - Lack of technical expertise, especially at local levels. - Adaptation of products to local situations. |
| 10102 | N/A |
| 10103 | Below are some challenges that digital public goods for the SDGs could run into:   - National and commercial legal restrictions on the public dissemination of data. - Difficulties in data standardization, including in related industries. - Regional and social technical constraints and differences that reduce the global availability of digital public goods. - Lack of information about the validity of the data. - The need for moderation of public open data. |
| 10104 | The likely challenges include economic downturn, competing international agendas, and concern about information security. |
| 10105 | Digital public goods that support SDG work should be able to directly serve specific goals or indicator monitoring and evaluation products. These products are fair, easy to understand, and the production process can be replicated. Users only need to perform simple operations on digital public goods, and a good application effect can be obtained.  Challenges to face: the identification of intellectual property rights of SDG public goods; the identification of the results and their attribution based on the output of SDG public goods; the software and hardware facilities for the storage and distribution of digital public goods. |
| 10106 | On the one hand, individual rights and collective interests need to be assessed and safeguarded, and on the other hand, there may be a huge gap between data owners and those who lack them. A broad range of actions is needed globally to reduce the associated risks, including building the capacity of all countries, particularly the least developed countries, landlocked developing countries and small island developing states. |
| 10107 | Voluntary free sharing, allowing relevant institutions and personnel to use and realize its value, can be considered public good use. There might be conflicts of interest in industry sectors. |
| 10108 | (1) Digital public goods are a technical means to realize the SDGs, and a key channel for the further popularization of SDG work.  (2) The application of digital public goods is limited by digital capabilities, data quality, digital trust, and regional factors. |
| 10109 | N/A |
| 10110 | Data are scattered and locked in each sector/country. Efforts are duplicated and data maintenance (updates) will be necessary as time passes. Finding the desired data, or said data not being interoperable or not (adequately) documented are a few other issues. |
| 10111 | Digital gap, data ownership, and data sharing policies, as well as capacity development are some of the challenges digital public goods face. |
| 10112 | - Duplication: There will be a need to coordinate with other international efforts. One main challenge is that we develop a digital public good and that, without us knowing, another institution is developing something very similar (duplication). This could cause problems since the end-user might get confused if they need to choose to use “digital public good developed by A” or “digital public good developed by B”. - Difficult or complex to use: If the digital public good is not user friendly, it will then be difficult for others to adapt. - Bugs: It will be quite complex to test the “digital public good” in all environments and in all types of EO Big Data, but that will be required in order to ensure that the “digital public good” works as expected. |
| 10113 | (Answers submitted together with respondent 10106) |
| 10114 | - Data challenges: There are differences between multi-source, multi-scale, and multi-temporal data. - Algorithm challenges: There is a lack of universal algorithms. - Platform challenges: The difficulties of multi-platform functions, large differences in positioning, and lack of interconnection need to be addressed. - Application challenges: What is the model for digital public goods to support SDG demonstration applications? |
| 10115 | Digital public goods need to have the characteristics of wide coverage, universality, high quality, easy access, ease of use by all people, and participation by all people.  The challenges we face include security guarantees, quality reviews, and soundness of the sharing mechanisms for digital public goods. |
| 10116 | N/A |
| 10117 | (Answers submitted together with respondent 10118) |
| 10118 | One problem is the willingness to share open data and code. Often data and source code are closed, which will hinder their wide utilization and future development |
| 10119 | Production and standardization of datasets are two challenges. |
| 10120 | (1) One of the challenges is to understand the concept and connotation of SDGs and digital public goods by participants or contributors. (2) The second challenge is to formulate qualitative standards and norms for digital public goods that can support SDG work. |
| 10121 | We should first determine its priority, then score its performance under different SDG targets, and finally evaluate it, while facing the challenges of unclear scoring mechanisms and vague product positioning. |
| 10122 | (1) One of the challenges is to understand the concept and connotation of SDGs and digital public goods by participants or contributors. (2) The second challenge is to formulate qualitative standards and norms for digital public goods that can support SDG work. |
| 10123 | Establish norms for data use and sharing, and characterize digital public goods that support SDG work. The challenge is the form and source of data sharing. |
| 10124 | The characteristics of digital public goods that can support SDG work should include: the characteristics of digitization, networking, and collaboration.  The challenges are: (1) how to mine and analyze these data to evaluate and improve SDG indicators; (2) integrating and mining data requires continuous investment; (3) decentralization and the iterative update of digital public goods that can only be realized by classifying the flow of decision-making, contributions, and information. |
| 10125 | The most likely challenges I foresee are: (1) whether SDGs can be fully understood; (2) data governance. |
| 10126 | Digital public goods can be characterized from the perspective of whether they can provide or reproduce information related to SDGs. However, due to the large number of similar related products, how to choose the best digital public goods will be a challenge. |
| 10127 | N/A |
| 10128 | 1) How to transform scientific data into general data products that can be interpreted and applied by decision-making departments and ordinary people. 2) How to accurately describe the background and prerequisites for the construction of digital public goods and define their application scenarios. 3) Data privacy, data flow mechanism, and data standards. 4) Data security, quality review, and sound sharing mechanisms. 5) Whether the product can be quickly obtained by global users; digital public goods, such as Google Earth, are interoperable. |
| 10129 | Likely challenges are as follows:   - Cross-border legislation. - Spatiotemporal variations:   - Geopolitical.   - Socioeconomic.   - Cultural variations. |
| 10130 | Political, social, and governance challenges will need to be addressed. |
| 10131 | - Balancing the development of flexible and standardized resources and data frameworks within the context of specific requirements of each SDG in each area. - Willingness to share data. Spatial data relies on data sharing as the location of core datasets (roads, buildings, land cover, etc.) is a resource almost anyone can use, so it would be most efficient to share these. |
| 10132 | Developers want to deliver, move fast, and often create barriers for their own tools and for our data. Therefore, not only training but real knowledge transfer and change is needed. |
| 10133 | In terms of data characteristics, it should have sufficiently diverse collection content, and collect as much data as possible from various countries in the world and in different dimensions. Sufficient support for comparative analysis of diversity is needed. At the same time, due to regional and political factors, it will be difficult to obtain information and identify forgery. |
| 10134 | It should be no small challenge to popularize these activities and let everyone actively participate. |
| 10135 | The main challenges are the digital infrastructure gap and capacity building.  The 2030 Agenda should also focus regionally as well as continentally instead of individual states. |
| 10136 | I agree. The implementation of digital public goods for SDGs carries challenges to solve existing problems, such as a lack of data and technological barriers to the implementation of the SDGs, through methods like data sharing, technological solutions, decision-making support, as well as capacity building for developing countries.  However, in my opinion, this phrase applies for all countries (not only for developing ones)—all stakeholders can find common ground.  Additionally, I suggest utilization of crowdsourcing to consider as a challenge for SDGs.  The crowdsourcing participants often are volunteers and independent citizen-stakeholders who provide their digital public goods (e.g., identifying, positioning, and qualifying waste-heaps) realizing cross-monitoring or helping to watch the environment—this makes a good challenge as well. |
| 10137 | Data are not readily available or the definition is not clear. |
| 10138 | The challenges are the same as for other digital research public goods, including protection of national security, proprietary concerns, personal privacy, research integrity, etc. These should be minimized as much as possible. |
| 10139 | The political environments and pandemics in the world affect the collaboration of scientists and international organizations and cause uncertainty in implementing digital public goods for SDGs. |
| 10140 | In the sharing platform, establish standards and norms for the production, sharing, and release of digital public goods, and use forms such as "giving bonuses, certificates, or publicizing the list of contributors" to increase the enthusiasm of contributors to digital public goods. |
| 10141 | From the perspective of data reliability and accuracy, it faces challenges such as data credibility. |
| 10142 | Digital public goods that can support SDG work should be innovative and valuable. It should be characterized by whether it is innovative and valuable. The challenge is that the qualitative criteria are relatively broad and difficult to quantify. |
| 10143 | According to the SDG guidance documents, accuracy, credibility, and national recognition are all challenges. |
| 10144 | N/A |
| 10145 | Establish a professional and responsible institution (or mechanism) to carry out qualitative work on digital public goods on the basis of compliance with national laws and regulations. The main challenge lies in the timeliness and accuracy of data products. |
| 10146 | Government authorities are passive about opening data. Laws and regulations are not specific enough, and there are certain risks in the sharing and opening of data/information. There is a lack of public platforms and sharing channels are not smooth. |
| 10147 | Digital public goods that can support SDG work must first ensure the reliability and safety of product quality, whether the scale is global or regional, and the continuous updating of data should be maintained with the characteristics of standardization, security, and reliability.  The challenges faced by digital public goods that support SDG work mainly include data reliability and continuous update capabilities. |
| 10148 | Lack of continuous updates of the public goods will need to be fixed. |
| 10149 | Qualitative analysis is made from the perspective of the necessity and availability of digital public goods. |
| 10150 | Challenges may include internal links between digital public goods, which cannot be well expressed by mathematical models. |
| 10151 | Understanding the creation/background and elements of public goods is the main challenge. |

| **Question 6** \| What approach should be in place to cope with rapid changes in the digital landscape while ensuring widescale adoption of any digital public good for SDGs? |
| --- |

| Respondent # | Response |
| --- | --- |
| 10101 | N/A |
| 10102 | N/A |
| 10103 | An agile approach should be used in the planning and implementation of any digital public good, responding not only to rapid technical changes, but also adapting to regional and social aspects. It is also important to create tools for rapid response to natural and social catastrophic events, when digital public goods can be generated and distributed under special conditions. |
| 10104 | A bottom-up community approach should be in place to supplement a top-down and resource- and technology-focused approach. |
| 10105 | Establish an open application mechanism for digital public goods or launch a global application plan, increase the intensity of multi-field cross-cooperation to carry out the application of SDG-specific goals or indicator monitoring and evaluation, and comprehensively promote the continuous update and iteration of digital public goods driven by demand. |
| 10106 | Make effective use of next-generation information technologies such as the Internet of Things, blockchain, artificial intelligence, and cloud platforms for big data processing to achieve fast and efficient processing of multi-source spatiotemporal heterogeneous big data, and complete the digital transformation to ensure the widespread adoption of any digital public goods for the SDGs. |
| 10107 | Understand cutting-edge trends and keep up with the needs of development and the demand from institutions that will apply the technology. |
| 10108 | Multi-platform common sharing, easy to upgrade and use. |
| 10109 | Improve the availability of public goods and strengthen basic theoretical research. |
| 10110 | Agile approaches are probably a good way to rapidly adapt to the fast-changing digital landscape. |
| 10111 | Tailor-made basic education and provision of user-oriented training programmes for continuing learning opportunities should be included in future policy. |
| 10112 | The “digital public good” will be required to be as open as possible and as independent from software/hardware as possible. We will need to set up a good basis of end-users who will then share any improvements or updates that they have developed in order to improve one of our “digital public goods”. |
| 10113 | (Answers submitted together with respondent 10106) |
| 10114 | Carry out data fusion and integration, enhance data acquisition and observation, and form high-quality, dynamically updated digital public goods; develop universal algorithms to ensure the stability of digital public goods production; integrate multi-scenario application models, and form stable service capabilities. |
| 10115 | Adopt mature, stable and reliable technology to produce, share and apply public goods to ensure the high quality and high availability of public goods.  Keep up with the development trend of science and technology, use advanced technology to continuously update digital public goods, and expand the application fields and methods of digital public goods. |
| 10116 | N/A |
| 10117 | (Answers submitted together with respondent 10118) |
| 10118 | Form big expert pools and provide tools to create and enhance unity between sections/groups/fields. These could be digital or in-person platforms. |
| 10119 | What is needed is a participatory approach involving all actors. |
| 10120 | It is necessary to establish a continuous mechanism to ensure the regular update and application of public goods, so as to ensure that the latest technology can serve public goods. |
| 10121 | Regular maintenance, demand-oriented goods, and products that are scalable should be emphasized. |
| 10122 | Ensure the establishment of acquisition, application, and user ecology of public goods. |
| 10123 | Actively promote and advocate for data sharing. |
| 10124 | Some technology companies use artificial intelligence technology to creatively provide technical solutions to many social pain points that were difficult to solve through traditional methods before, forming a number of influential brand projects, such as Geely's "Ji Shi Yu" and JD.com's " "Things Love Connected", Ali's "Ant Forest", Tencent's "99 Public Welfare Day", Baidu's "AI Finding People" and so on. These projects have enabled public goods to be widely used and created new business models.  Therefore, we should not only fit the SDG goals in the topic selection of digital public goods, but also pay attention to the actual social pain points and people's livelihood, so that digital public goods can effectively use the power of technology to deal with current social problems, so as to be widely used. |
| 10125 | Data security and application security will be the main focus of any approach. |
| 10126 | To maintain the long-term vitality and wide application of digital public goods, reasonable standards for production should be formulated from the very beginning, with timely updates and continuous optimization according to technological development and data sharing. |
| 10127 | N/A |
| 10128 | We need the support of a technology platform. By building a scalable "cloud" technology support platform, it integrates algorithms and collects data. Data standardization, high quality assurance. The update of the data product itself must meet the needs of users for the product, and at the same time, the data platform/operating system/development platform supported by the product must keep up with the pace of the times. |
| 10129 | Any approach should consider:  1) Interdisciplinarity.  2) Initiating and enforcing cross-border legislations, as in the EU examples.  3) Enhancing academia-industry partnerships. |
| 10130 | The application of a (geoscience) digital ecosystem approach (at different levels) and the definition of new digital resource sharing and governance acts (at regional and international levels) are key points. |
| 10131 | Dedicated resources with flexibility. It is important for international cooperative frameworks to have dedicated resources to quickly respond to emerging technology and test innovative frameworks. It is also important to allow for flexibility and openness of local and regional users to pick up only the parts of the framework that work for them. |
| 10132 | FAIR D.O. |
| 10133 | It should be ensured that public welfare products have sufficient and durable self-renewal capabilities. Secondly, to deal with rapid iterations in the field of science and technology, it is necessary to receive the latest technical solutions in a timely manner, deepen and improve their own content construction in a targeted manner, and ensure the stability of their core functions. On the basis of rapid expansion, a successful product will increase the application breadth of public goods, and gain more supporters and developers to invest in it. |
| 10134 | It needs to be strategically forward-thinking at the beginning of the design, leaving some advanced interfaces, and the platform can be iterated after the technology is updated. |
| 10135 | You will need a global strategy of adaptation. This means for the case of Africa and Arab States, the African Union (AU) and League of Arab States (LAS) should be involved. Both AU and LAS have agendas to implement the UN 2030 Agenda. However, they have not heard of Open Science/Open Data or GOSC. They have a digital infrastructure gap which hinders their ability to process big data upon which the SDG’s are based. GOSC will ensure that rapid changes in technology are quickly assimilated. |
| 10136 | Remote sensing satellites together with in-situ data collections give us the best answer. Here the expenses must be well-balanced.  Rapid changes in the digital landscape can be detected mainly locally, provided the drone technology does well in rapid monitoring, of course, together with in-situ data collections (professional or crowdsourcing). This possibility can be utilized when the drone-operators and other actors are well networked for a digital public goods type approach. |
| 10137 | Make the data platform open-access and everyone can contribute to the updates of changes. The core algorithm should be suited to varying variables, though. |
| 10138 | Establishment of active, independent, international expert committees that help guide and evaluate key SDG and digital infrastructure issues and activities should be included in the approach. |
| 10139 | In response to the UN’s call on the “Global Digital Compact”, all communities need to strengthen collaborations in different ways in the digitally transforming world to ensure widescale adoption of any digital public good for SDGs. |
| 10140 | Carry out extensive publicity actions on the sharing platform, encourage users to actively apply public welfare products, and provide materials such as application maps, application results, and application effect certificates. Utilize incentives such as "giving bonuses, certificates or publicizing the list of contributors" to increase the enthusiasm of users of digital public goods. |
| 10141 | Strengthen the publicity of public goods and encourage more people to share data and models. |
| 10142 | Timely publicity should be implemented, and at the same time, the public goods product itself must have greater use value. |
| 10143 | It can really play an important role and be recognized by the corresponding national business department if planned properly. |
| 10144 | N/A |
| 10145 | It is possible to learn from or introduce new technologies, find the point of convergence between them and digital public goods, combine the two, and apply them in the production, release, and application of digital public goods. |
| 10146 | Try to choose some classic and common public goods. In addition, the public goods themselves need to be constantly updated and innovated. |
| 10147 | In view of the rapid iteration in the field of science and technology, public goods need to keep pace with the times to track the development of disciplines, widely apply multi-source data or new data, maintain continuous updates and iterations of spatiotemporal resolution, improve the calculation and production efficiency of public goods, and provide data products, algorithms, models, and digital platform update capabilities to expand applications. |
| 10148 | Cloud platforms for data analysis could be effectively utilized in an approach to update digital public goods. |
| 10149 | Strive to make digital public welfare products irreplaceable, authoritative, forward-looking, and compatible. |
| 10150 | Define the application fields of public goods and update public goods in combination with scientific and technological development. |
| 10151 | Ensure the establishment of access, application, and users of public goods. |

| **Question 7** \| Do you see potential in strengthening multi-stakeholder digital cooperation within the framework of digital public goods for SDGs? |
| --- |

| Respondent # | Response |
| --- | --- |
| 10101 | Yes, and this is very important. There are already such actions being taken within the UN initiative under ITU. |
| 10102 | Yes. |
| 10103 | <https://firms.modaps.eosdis.nasa.gov>  Providing Active Fire Data for Near-Real Time Monitoring and Applications  The Fire Information for Resource Management System (FIRMS) distributes Near Real-Time (NRT) active fire data from the Moderate Resolution Imaging Spectroradiometer (MODIS) aboard the Aqua and Terra satellites, and the Visible Infrared Imaging Radiometer Suite (VIIRS) aboard S-NPP and NOAA 20 (formally known as JPSS-1). Globally these data are available within 3 hours of satellite observation, but for the US and Canada active fire detections are available in real-time.  https://apgc.awi.de/about  The Arctic Permafrost Geospatial Centre (APGC)  APGC is an Open Access Circum-Arctic Geospatial Data Portal that promotes, describes and visualizes geospatial permafrost data. A data catalogue and a WebGIS application allow to easily discover and view data and metadata. Data can be downloaded directly via link to the publishing data repository. |
| 10104 | There is definitely potential in strengthening multi-stakeholder digital cooperation, when digital public goods can enhance availability, accessibility, acceptability and quality to digital resources and technologies for more, if not all. |
| 10105 | Encourage the sharing of digital public goods (data). Multi-party and multi-field experts should cross-cooperate to carry out demonstration applications of SDG-specific goals or indicator monitoring and evaluation, and provide access to technical support and assistance with digital public goods to underdeveloped countries, regions, and groups. |
| 10106 | Yes, under the SDG digital public goods framework, there is great potential to strengthen cooperation among governments, organizations, relevant groups, and other stakeholders, share relevant technical knowledge, information, and experience, and address common challenges. |
| 10107 | Cooperation is very necessary, especially with advantageous institutions in the industry and field. |
| 10108 | Yes. |
| 10109 | Yes. |
| 10110 | N/A |
| 10111 | Though multi-stakeholder cooperation always must bear transaction costs, it has to be recognized that no single programme nor agency is in the position to provide all sorts of digital public goods for SDGs. Therefore, the need for developing a cooperative framework with a participatory approach and full potentiality is envisaged, and building such a framework it should observe the principles of inclusive, participatory approaches, and follow the model of extensive consultation, joint contribution and shared benefits.  Within the general SDGs framework, a wide range of stakeholders could be aligned, including governments, international organizations, private sector, and NGOs. One such example is FAO, which has been providing a broad spectrum of digital public goods for agriculture and food security for decades. In particular, their recent efforts on global soil information mapping, global soil lab network, (GLOSOLAN), and recarbon soil initiative (RECSOIL) are good examples to be noted. |
| 10112 | Yes, if necessary, you can contact me. |
| 10113 | (Answers submitted together with respondent 10106) |
| 10114 | Yes. Especially in terms of demonstration applications, it is necessary to rely on the coordination and cooperation of all parties to clarify the actual needs and carry out targeted services. |
| 10115 | Yes, by using multi-platform and multi-channel publicity, establishing and improving the production and sharing mechanism of digital public goods, and building a public platform that is easy to use and easy to participate in. |
| 10116 | N/A |
| 10117 | (Answers submitted together with respondent 10118) |
| 10118 | Yes, I think this is the most important thing to make this goal a reality. I and INAR are willing to help as much as possible to implement big data sharing and utilization in a global context. |
| 10119 | Yes, cooperation at different spatial and organizational scales is a major asset. |
| 10120 | SDG work involves environmental, social, and economic aspects. It is comprehensive work, and it is very necessary to strengthen multi-party cooperation. |
| 10121 | Yes. The realization of the SDGs cannot be separated from digital cooperation based on shared values, which will involve multiple fields. |
| 10122 | Yes. SDG work involves environmental, social, and economic aspects. It is a comprehensive work, and it is very necessary to strengthen multi-party cooperation. |
| 10123 | It is necessary to strengthen multi-party cooperation to promote the application and digital public goods in the SDG field. |
| 10124 | Establish a mechanism for joint promotion of data product production, joint maintenance of data product security, joint participation in data governance, and joint sharing of data results to promote multi-party cooperation in digital public goods. |
| 10125 | It requires the government, scientific research institutes, universities, and enterprises to cooperate with each other. |
| 10126 | SDG work involves many fields, and the types of digital public welfare items that each field or unit can provide are relatively limited. To fully ensure the smooth development of SDG work, it is necessary to strengthen multi-party cooperation. |
| 10127 | Yes. |
| 10128 | Yes. It is necessary to promote data sharing, strengthen mutual exchanges between scientific research institutions and decision-making departments, strengthen cooperation between local universities and governments, and strengthen the export of digital products and services to developing countries internationally. |
| 10129 | Yes, given the technological trends, there is great potential in strengthening multi-stakeholder digital co-operation, particularly if countries become increasingly aware of the impact global phenomena have on us all. Establishing big data ecosystems, focusing on Big Earth Data, is one way the science community can practically demonstrate the role we have to play in salvaging our planet. This is because “our one earth” is universally affected by our actions. However, the exact answer to this question depends more on the political will than on anything else. |
| 10130 | Yes. |
| 10131 | Yes. As I stated above, geographers have high stakes in data sharing as we all use the location of items in space and then add our own data. For example, buildings and roads can be mapped by one organization, and then other organizations can add population census data, flood risk, or economic information to those locations. All of this is dependent on core spatial datasets—something HOT is utilizing OSM for. |
| 10132 | Yes, there is both potential and necessity. |
| 10133 | Carry out online and offline publicity activities, actively participate in major well-known conferences, and invite well-known experts to try out endorsements. At the same time, it is necessary not only to keep in close contact with related fields, but also to create suitable docking opportunities in other non-related fields. Explore potential cooperation possibilities. |
| 10134 | Yes. But at the beginning, we need a demonstration to let everyone participate and let the data flow. Then continue to strengthen multi-party cooperation. |
| 10135 | Yes. For example, AASCTC can be a regional stakeholder for Africa and Arab States. The planned Africa Arab Science and Technology Cloud (AAScTCloud) is based on GOSC and can give services for Africa and Arab States. This will close the digital infrastructure gap and also provide UN SDG 2030 services based on processing Big Data. Partnership between CBAS and AASCTC can help in coordinating all activities.  This will provide a central node for Africa and Arab States. AASCTC will create partnerships with AU and LAS as well as the UN Department of Economic and Social Affairs (DESA) and ITU. |
| 10136 | Digital public goods for SDGs promote the implementation of SDGs. Their efficiency will be highly increased by strengthening digital cooperation at the multi-stakeholder level (stakeholders as owners or users of the open goods). It is easily estimable. However, detailed knowledge on the circumstances between stakeholders, digital public goods, and tasks (aimed by goods) definitely contribute to high probability estimation of the strengthening/efficiency rate. |
| 10137 | Yes. |
| 10138 | Yes. See answers to #4 and 6 above. There should be a systematic review of all relevant organizations that need to be involved. |
| 10139 | Yes. I do. |
| 10140 | It is necessary to strengthen multi-party cooperation in the field of digital public goods supporting SDG work, and increase the visibility of digital public goods supporting SDG work! |
| 10141 | Yes, digital public goods in support of the SDGs require multi-party cooperation and joint promotion. |
| 10142 | Yes, on the following conditions:   - Increase support for partners. - Give full play to the leadership and decision-making role of the organization. - Strengthen institutional norms and organizational promotion. - Strengthen international cooperation. |
| 10143 | Yes. |
| 10144 | Yes. |
| 10145 | Absolutely yes. It is necessary. Taking SDGSAT satellite data as an example, through multi-party cooperation, the production of global digital public goods at multiple scales can be realized, and it will also help the public to understand Earth more deeply from different scales. |
| 10146 | Yes, especially the need to strengthen the support of the government and industrial sectors. |
| 10147 | Measures to promote multi-party cooperation in the field of digital public goods to support SDG work mainly include: establishing close ties with international organizations and platforms, providing support for public goods, and improving the influence of digital public goods; cooperation in the field of SDGs; carrying out digital public goods to support the progress or achievements in areas working toward the SDGs. |
| 10148 | Yes, international cooperation under a UN framework helps to achieve the goal. |
| 10149 | Yes. |
| 10150 | Yes. |
| 10151 | Yes, there is potential. |
